# Generalizable transcriptome-based tumor malignant level evaluation and molecular subtyping towards precision oncology

**DOI:** 10.1186/s12967-024-05326-0

**Published:** 2024-05-28

**Authors:** Dingxue Hu, Ziteng Zhang, Xiaoyi Liu, Youchun Wu, Yunyun An, Wanqiu Wang, Mengqi Yang, Yuqi Pan, Kun Qiao, Changzheng Du, Yu Zhao, Yan Li, Jianqiang Bao, Tao Qin, Yue Pan, Zhaohua Xia, Xin Zhao, Kun Sun

**Affiliations:** 1 Institute of Cancer Research, Shenzhen Bay Laboratory, Shenzhen, 518132 China; 2 Hepato-Biliary Surgery Division, The Second Affiliated Hospital, Shenzhen Third People’s Hospital, Southern University of Science and Technology, Shenzhen, 518100 China; 3 Department of Biology, Southern University of Science and Technology, Shenzhen, 518055 China; 4 Thoracic Surgical Department, Shenzhen Third People’s Hospital, The Second Affiliated Hospital, Southern University of Science and Technology, Shenzhen, 518100 China; 5 Department of Biochemistry, School of Medicine, Southern University of Science and Technology, Shenzhen, 518055 China; 6 Molecular Cancer Research Center, School of Medicine, Shenzhen Campus of Sun Yat-sen University, Sun Yat-sen University, Shenzhen, 518107 China; 7 Division of Life Sciences and Medicine, University of Science and Technology of China, Hefei, 230027 China; 8 Guangdong Provincial Key Laboratory of Malignant Tumor Epigenetics and Gene Regulation, Guangdong-Hong Kong Joint Laboratory for RNA Medicine, Medical Research Center, Sun Yat- Sen Memorial Hospital, Sun Yat-Sen University, Guangzhou, 510120 China; 9 Beijing Tsinghua Changgung Hospital, Tsinghua University School of Medicine, Beijing, 102218 China; 10 Integrative Microecology Clinical Center, Shenzhen Key Laboratory of Gastrointestinal Microbiota and Disease, Shenzhen Clinical Research Center for Digestive Disease, Shenzhen Technology Research Center of Gut Microbiota Transplantation, Shenzhen Hospital, Southern Medical University, Shenzhen, 510086 China

**Keywords:** Oncogene, Tumor suppressor, Survival analysis, Hepatocellular carcinoma

## Abstract

In cancer treatment, therapeutic strategies that integrate tumor-specific characteristics (i.e., precision oncology) are widely implemented to provide clinical benefits for cancer patients. Here, through in-depth integration of tumor transcriptome and patients’ prognoses across cancers, we investigated dysregulated and prognosis-associated genes and catalogued such important genes in a cancer type-dependent manner. Utilizing the expression matrices of these genes, we built models to quantitatively evaluate the malignant levels of tumors across cancers, which could add value to the clinical staging system for improved prediction of patients’ survival. Furthermore, we performed a transcriptome-based molecular subtyping on hepatocellular carcinoma, which revealed three subtypes with significantly diversified clinical outcomes, mutation landscapes, immune microenvironment, and dysregulated pathways. As tumor transcriptome was commonly profiled in clinical practice with low experimental complexity and cost, this work proposed easy-to-perform approaches for practical clinical promotion towards better healthcare and precision oncology of cancer patients.

**Supplementary Information:**

The online version contains supplementary material available at 10.1186/s12967-024-05326-0.

## Introduction

Cancer is the second leading cause of mortality globally, responsible for as many as 10 million deaths every year. In China, there are 4.8 million newly diagnosed cases and 3.2 million deaths per year [1]. Carcinogenesis is a very complex process with many genes and pathways dysregulated, which leads to high heterogeneity among tumors (including those of the same cancer type) [2]; hence, unraveling the genetic heterogeneity of tumors is widely considered to be pivotal in precision oncology, especially for guiding patient-specific targeted therapies to improve clinical outcomes [3]. Various genes that are considered particularly important in cancers have been identified, and most of them are classified as oncogenes or tumor suppressors based on their molecular functions in tumorigenicity and tumor progression [4]. However, the current catalogue of such cancer-associated genes is far from being complete, and the nexus between molecular landscape of these genes in tumors (i.e., basic science) and guidance for precision oncology of cancer patients (i.e., translational medicine) also requires more comprehensive investigations and explorations [5].

In clinical practice, the tumor malignant level and subtypes are highly informative in guiding the therapeutic interventions of the patients. Conventional methods to evaluate the tumor malignant level mostly rely on tumor size, number of affected lymph nodes and metastatic status (i.e., TNM staging). For example, the Barcelona Clinic Liver Cancer (BCLC) staging system is the most widely used approach for Hepatocellular carcinoma (HCC) [6, 7]. BCLC system is considered informative for optimizing treatment strategies in early-stage HCC patients, while its guidance for advanced-stage patients is limited [8–10]. One shortage of current staging systems is that the molecular characteristics of the tumors are not fully utilized [11]. In fact, tumors at the same stage may possess drastically distinct biological traits that impact the responses to drugs [12], hence, investigations on the molecular profiles of the tumors are of high clinical potential. For example, breast cancer can be categorized into four subtypes based on the gene expression patterns, each exhibiting distinct prognostic and therapeutic responses [13, 14]. However, clinically validated molecular subtyping methods are only available for a limited number of cancer types. In particular, HCC exhibits both high morbidity and mortality but without efficient subtyping approaches; moreover, considering that Chinese HCC patients frequently suffer from Hepatitis B Virus infections while alcoholic liver disease is more common in western countries [15], such essentially different genetic background makes HCC subtyping more challenging. Recently, various HCC subtyping approaches based on proteogenomic landscape [16], gene mutation [17], non-coding RNA [18], and immune signatures [19] had been proposed, while the experimental complexity and high cost had adversely affected large-scale validation and promotion of these methods. Thus, it is of urgent demand to develop simple and effective approaches to meet the clinical needs.

In previous studies, we and others have demonstrated that the transcriptomes contain massive molecule information of tumors with high clinical values [20–22]. For instance, we showed that using transcriptome data from tissue biopsies alone, one could predict its malignant status, evaluate its purity, as well as predict its tissue origin with very high accuracy [23, 24]; Song et al. proposed that gene expression is sufficient to catch the major biological discrepancies among different subgroups in pancreatic cancer [25]. More importantly, as transcriptome profiling is inexpensive and easy-to-perform (e.g., through RNA-seq), it has been widely used in clinical practice, for example, to screen for known druggable targets. Therefore, in this study, we further explored the translational value of tumor transcriptome, including investigations on key genes in cancers as well as developments of tumor malignant level evaluation and subtyping approaches.

## Results

### Various types of prognosis-related genes across cancers

**Fig. 1:**
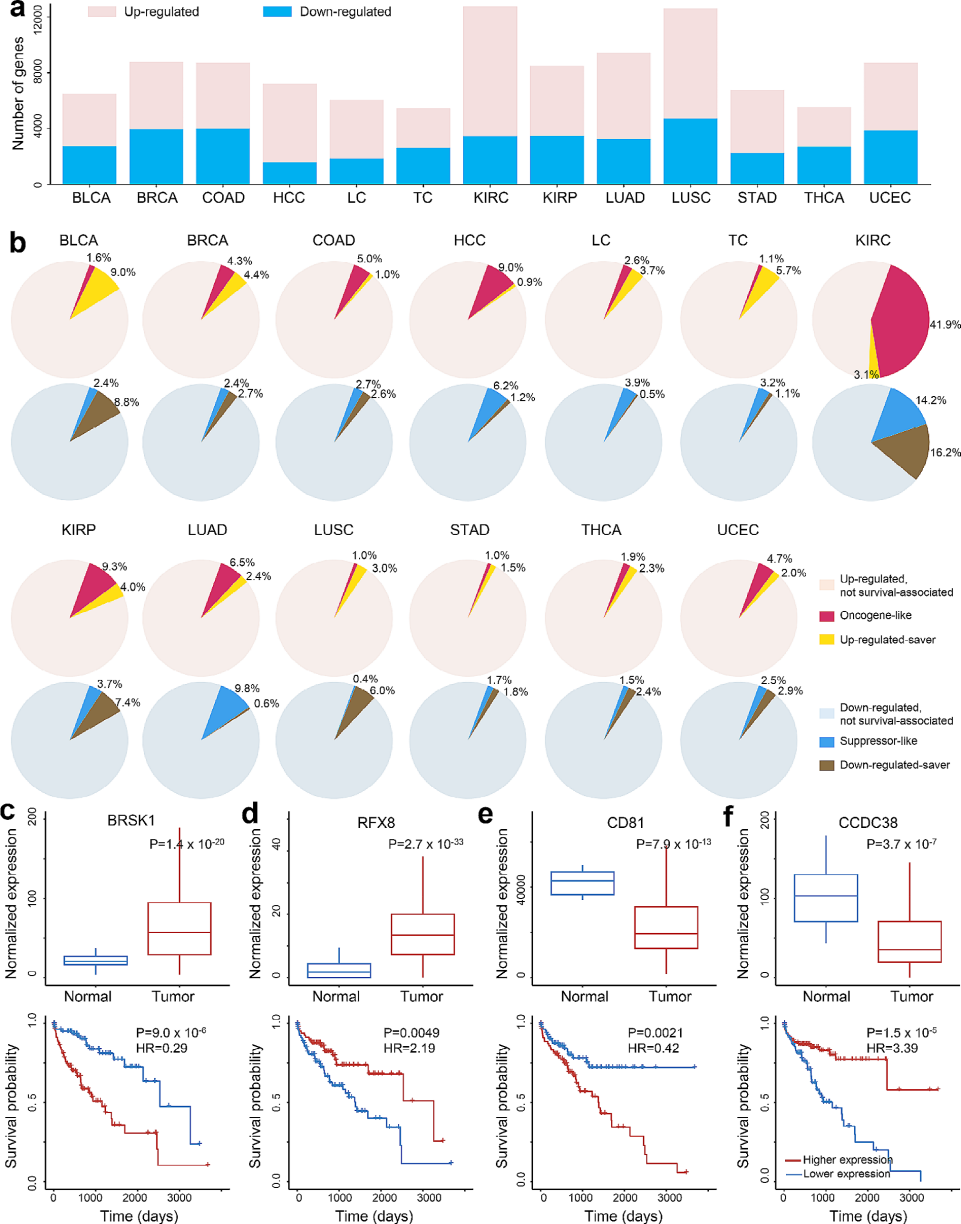
Identification of dysregulated and prognosis-associated genes across cancers. (**a**) Frequency of differentially expressed genes; (**b**) The associations of the gene expression with prognosis. The dysregulated and prognosis-associated genes were categorized into 4 groups with illustrations using genes in hepatocellular carcinoma (HCC): (**c**) oncogene-like, (**d**) up-regulated-saver, (**e**) down-regulated-saver, and (**f**) suppressor-like. For c-f, the upper panels showed normalized gene expression levels (by DESeq2) between tumor (red) and adjacent normal (blue) samples, and the adjusted p-values (P) were calculated by DESeq2. The lower parts showed the prognosis of patients divided into 2 groups: red and blue curves stand for patients with relatively higher and lower expression, respectively. P-values and Hazard Ratios (HR) were calculated using Cox regressions

We collected RNA-seq data and patients’ prognosis information from The Cancer Genome Atlas (TCGA) [26]. As we were mostly interested in differential expression and prognosis analysis, only the cancer types with at least 10 adjacent normal samples and 100 tumor samples were selected (*N* = 13, Suppl. Tables S1-S2) for achieving statistical power in downstream analyses: Bladder urothelial carcinoma (BLCA), Infiltrating duct carcinoma of the Breast (BRCA), Colon adenocarcinoma (COAD), HCC, Laryngeal cancer (LC), Tongue cancer (TC), Kidney renal clear cell carcinoma (KIRC), Kidney renal papillary cell carcinoma (KIRP), Lung adenocarcinoma (LUAD), Lung squamous cell carcinoma (LUSC), Stomach adenocarcinoma (STAD), Thyroid carcinoma (THCA), Endometrial endometrioid adenocarcinoma (UCEC). For each cancer type, we mined the genes that were differentially expressed (DEGs) in the tumors compared to adjacent normal samples (Suppl. Fig. S1). As shown in Fig. 1a, the number of DEGs varied drastically, ranging from 5,511 in TC to 12,736 in KIRC. Then, for each DEG in each cancer type, we divided the tumor samples into 2 categories based on the median normalized expression of this DEG in the corresponding cancer type (i.e., samples with expression values of the DEG higher than the median were defined as “higher-expression” category, and the rest samples were “lower-expression” category) and performed prognosis analysis between these two categories. As a result, we found that only a small proportion of DEGs (< 10% in most cancer types) were associated with patients’ survival (Fig. 1b). Interestingly, for DEGs that were up-regulated in tumors, higher expression (i.e., higher than median expression level in all the tumors) did not always link to poorer survival but could correlate with better survival of patients; similarly, lower expression (i.e., lower than median expression level in all tumors) of DEGs that were down-regulated in tumors could also associate with better survival (Fig. 1c-f). Hence, we categorized the prognosis-associated DEGs into 4 groups: “oncogene-like”, “up-regulated-saver”, “down-regulated-saver” and “suppressor-like”. For any gene that was up-regulated in tumors, if patients with relatively higher expression showed worse prognosis, then these genes could be categorized as “oncogene-like”, such as *BRSK1* (Fig. 1c); in contrast, if higher expression led to better prognosis in cancer patients, such as *RFX8* (Fig. 1d), then this gene would be categorized as “up-regulated-saver”. For any gene that was down-regulated in tumors, if patients with lower expression showed better survival, this gene would be categorized as “down-regulated-saver”, such as *CD81* (Fig. 1e), otherwise, it would be categorized as “suppressor-like”, such as *CCDC38* (Fig. 1f). Distributions of these 4 types of DEGs varied significantly among cancer types. For example, for the down-regulated and prognosis-associated genes, most of them were “suppressor-like” in LUAD; as a contrast, in LUSC, “down-regulated-saver” was the majority. The data thus revealed intricated relationships between gene dysregulation and patients’ survival, which could be worthwhile for further explorations.

### Revisiting oncogenes and tumor suppressors

**Fig. 2:**
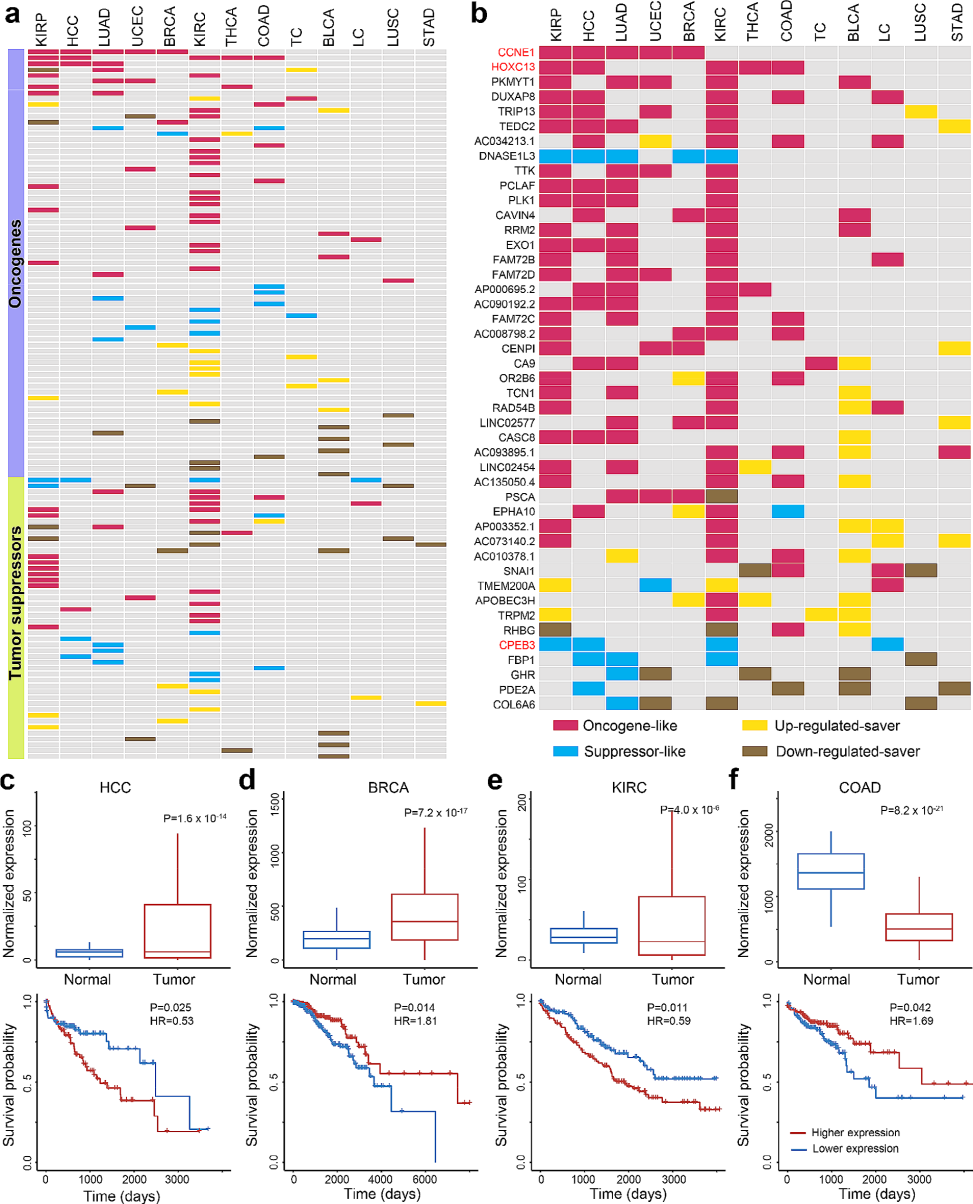
Prognostic characteristics of key cancer-related genes across cancers. (**a**) Genes in NCG database showing dysregulation and prognosis-association. (**b**) Genes frequently dysregulated and associated with prognosis. Genes highlighted in red (*N* = 3) are also reported in the NCG database. (c-f) Illustration of the expression and survival association of *EPHA10* gene in (**c**) HCC, (**d**) BRCA, (**e**) KIRC, and (**f**) COAD. For figure c-f, the upper panels showed normalized gene expression levels (by DESeq2) between tumor (red) and adjacent normal (blue) samples, and the adjusted p-values (P) were calculated by DESeq2. The lower panels showed the prognosis of patients divided into 2 groups: red and blue curves stand for patients with relatively higher and lower expression, respectively. P-values and Hazard Ratios (HRs) were calculated using Cox regressions

We then explored genes that were frequently associated with prognosis across cancers, as these genes might be highly related to cancer. We first investigated known cancer-related genes in the Network of Cancer Genes (NCG), a high-quality manually curated database for oncogenes and tumor suppressors [27]. The results were summarized in Fig. 2a and Suppl. Table S3. Surprisingly, among the 256 oncogenes annotated in NCG, only 73 (28.5%) were found to be differentially expressed and associated with prognosis in at least 1 cancer type; moreover, only 38 (52.1%) showed “oncogene-like” behavior in at least 1 cancer type, while 35 (47.9%) showed inconsistent dysregulation and prognosis associations. For instance, *HLF* gene was down-regulated in COAD and LUAD, and in both cancers, lower expression was associated with worse survival of the patients (i.e., it was “suppressor-like”). Similarly, among the 251 tumor suppressor genes annotated in NCG, only 48 (19.1%) were found to be differentially expressed and associated with prognosis in at least 1 cancer type; moreover, only 12 (25.0%) showed “suppressor-like” behaviors in at least 1 cancer type, while 36 (75.0%) showed inconsistent prognosis associations. Moreover, the number of prognosis-associated NCGs varied among cancer types, showing higher frequencies in kidney cancers while lower in digestive system cancers, e.g., STAD and LC.

We further explored potentially novel cancer-related genes using a threshold of showing prognosis association in at least 4 cancer types (i.e., > 30% in all cancer types investigated). As a result, 45 genes showed up. Interestingly, only 3 of them (*CCNE1*, *HOXC13*, *CPEB3*) existed in the NCG database (Fig. 2b and Suppl. Table S4). The majority (77.8%) of these putative cancer-related genes showed “oncogene-like” behaviors among various cancer types. For example, *PKMYT1* showed “oncogene-like” behavior in 5 cancer types, and *DNASE1L3* recurrently showed “suppressor-like” behavior in 5 cancers. On the other hand, 27 genes showed diversified behaviors among cancer types. *EPHA10* gene was illustrated in Fig. 2c-f as an example: it was annotated as an “oncogene” in NCG and did behave as “oncogene-like” in HCC and KIRC, but it also behaved as “up-regulated-saver” manner in BRCA and “suppressor-like” in COAD. The results echoed the dysregulation and prognosis-association patterns of genes annotated in NCG, demonstrating the complexity of cancer-related genes in affecting prognosis and suggesting further investigations on such genes in a cancer type-dependent manner.

### A universal approach for modeling tumor malignant level

**Fig. 3:**
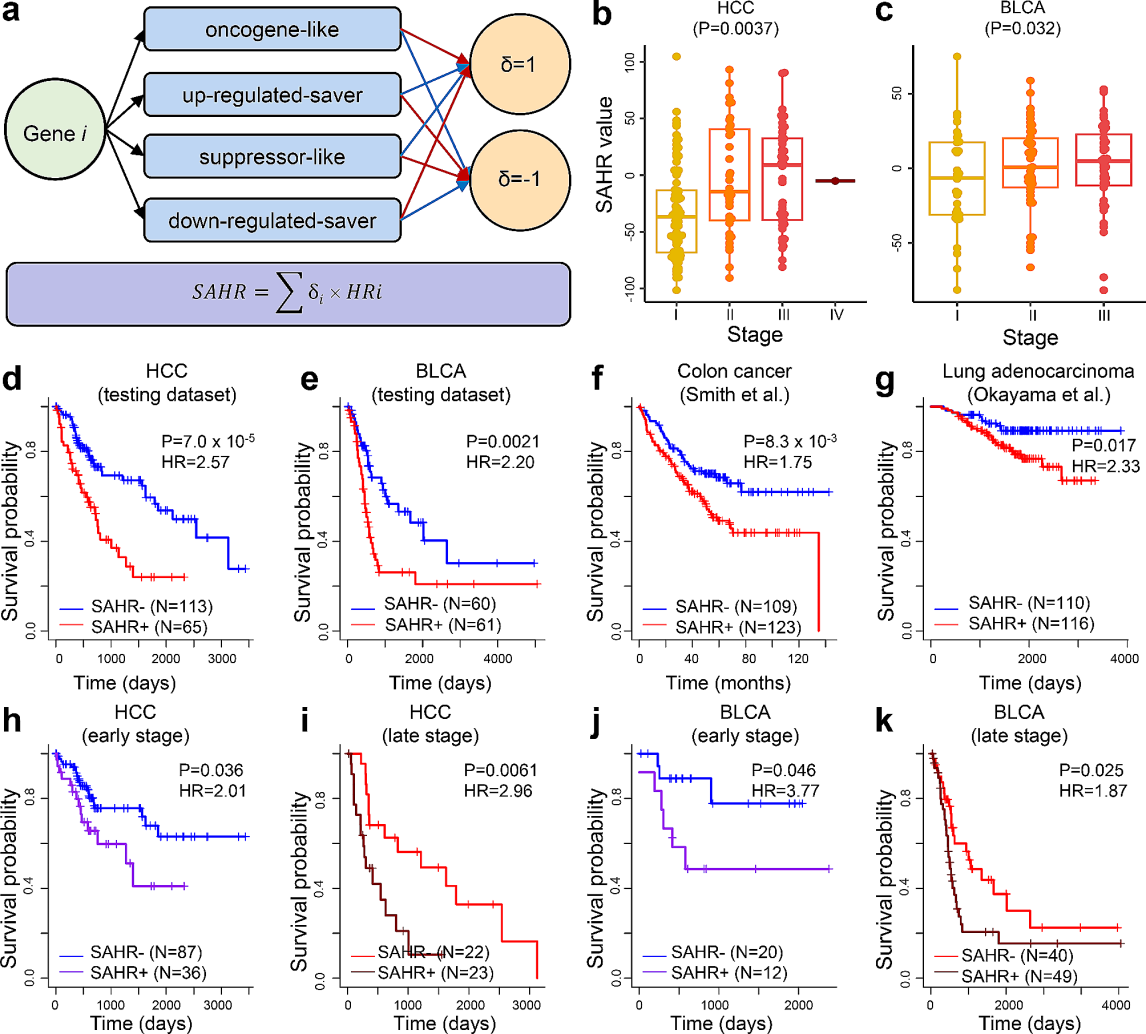
The SAHR (Score of Aggregated Hazard Ratio) model for tumor malignant level evaluation. (**a**) Formula for calculating SAHR for each sample to evaluate. Genes in 4 categories were used to build the model; for oncogene-like and down-regulated-saver genes, if the expression in the given sample was higher than the cutoff, then a coefficient of “+1” would be assigned to the corresponding Hazard Ratios (red arrow), otherwise a “-1” would be used as the coefficient (blue arrow); for up-regulated-saver and oncogene-like genes, if the expression in the given sample was higher than the cutoff, then a coefficient of “-1” would be assigned to the corresponding Hazard Ratios (HRs; red arrow), otherwise a “+1” would be used as the coefficient (blue arrow). The HRs of the genes were multiplied by their coefficients and then accumulated as the SAHR value. (**b**, **c**) Distribution of SAHR values among different clinical stages in (**b**) HCC and (**c**) BLCA. P-values were calculated using Kruskal-Wallis tests. (**d**) Comparison of patients’ survival with different SAHR values in the testing datasets of HCC and (**e**) BLCA. (**f**, **g**) Comparison of patients’ survival with different SAHR values in non-TCGA datasets: (**f**) colon cancer and (**g**) lung adenocarcinoma. The SAHR scores were calculated using models trained from TCGA’s COAD and LUAD samples, respectively. (**h**-**k**) Comparison of patients’ survival with different SAHR values in the early- (i.e., I and II) and late-stage (i.e., III and IV) samples in HCC and BLCA. P-values and HRs were calculated using Cox regressions. SAHR- and SAHR + stood for tumors with negative and positive SAHR values, respectively

Based on the profiles of prognosis associated genes revealed in Figs. 1 and 2, we wondered whether the expression patterns of these genes in a specific tumor could inform its malignant level (within the context of stand-of-care treatment). To do this, for each cancer type, we randomly split the tumor samples into 2 groups, 1 for training and the other for testing; for each DEG in each cancer type, we divided the samples in the training dataset into “higher-“ and “lower-expression” categories based on the median normalized expression of this DEG and performed survival analysis between these two categories; then, we picked up the DEGs that were most significantly associated with prognosis and collected the corresponding hazard ratios between higher- and lower-expressed samples; lastly, for each tumor sample, we calculated a parameter, named SAHR (Score of Aggregated Hazard Ratio) that used the expression levels as “coefficients” to aggregate the hazard ratios (Fig. 3a-c, Suppl. Fig. S2-S13). For example, for one gene classified as “oncogene-like”, if its expression in the tumor-of-interest was higher than the median value of all training samples, it would be assigned a coefficient of “+1” (red arrow in Fig. 3a), otherwise, it would be assigned a coefficient of “-1” (blue arrow in Fig. 3a). Under such settings, we hypothesized that higher SAHR value should indicate a higher malignant level of the tumor, and vice versa. Indeed, we found that in all cancer types, tumors with positive SAHR values were associated with significantly worse survival of the corresponding patients, as observed in the training datasets and validated in the testing datasets (Fig. 3d-e and Suppl. Fig. S3-S13). For cancer types with relatively large number of samples in the testing dataset, we found that SAHR models work well on patients with different genders and ethics (Suppl. Fig. S14). To further validate the SAHR models, 2 non-TCGA datasets were collected: 1 colon cancer dataset containing 232 samples from Smith et al. [28], and 1 lung adenocarcinoma dataset containing 226 samples from Okayama et al. [29]. Of note, transcriptome profiling in these two datasets was both performed using Affymetrix Human Genome U133 Plus 2.0 Array platform, which is different from TCGA. For each dataset, we first picked up the genes used in the corresponding SAHR model (COAD or LUAD) that were also profiled in the microarray platform; we then extracted the median expression values in the tumor samples as cutoffs to determine “higher-” and “lower-expression” category of the tumors and used the same HRs in the corresponding SAHR model (Suppl. Table S6) to calculate SAHR scores for each sample. Despite the big difference in data generating platform, highly consistent results to that on TCGA datasets were obtained: patients with negative SAHR values still showed significantly better survival than those with positive SAHR values (Fig. 3f-g), demonstrating the robustness and generalizability of our SAHR models.

Considering that the clinical stage is the most widely used parameter in evaluating the tumor malignant level, we further explored whether our SAHR model could add value to the clinical stage. In general, tumors with advanced stages tended to show higher SAHR values, while statistical significance was only observed in HCC, KIRP, KIRC, and LUAD (Fig. 3b-c, Suppl. Fig. S2). In addition, high variation in SAHR values from tumors of the same stage was observed, so we explored the correlations of SAHR values with prognosis of patients in the same stage. Due to the limited sample size of the testing datasets in most cancer types, we divided the samples into early- (i.e., I and II) and late-stages (i.e., III and IV) for each cancer type. The results for HCC and BLCA were shown in Fig. 3h-k and other cancer types can be found in Suppl. Fig. S3-S13: for both early- or late-stages in most cancer types, samples with positive SAHR values still showed worse survival than those with negative SAHR values in the same stage. These results demonstrated the translational significance of our SAHR model as a universal approach to measuring the malignant level of tumors from the molecular side of view.

### Transcriptome-based subtyping of HCC

**Fig. 4:**
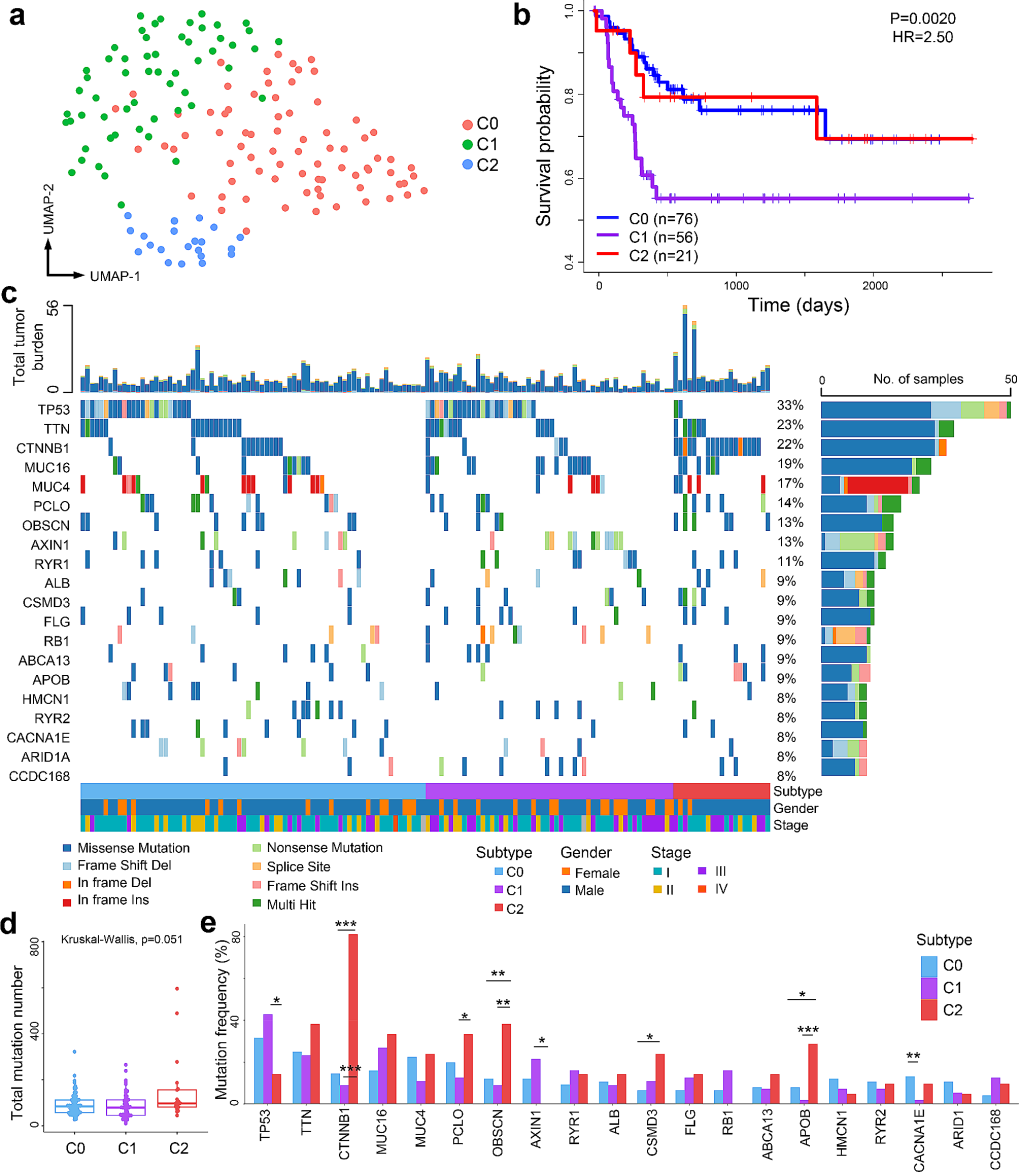
Transcriptome-based subtyping of HCC in Asian patients. (**a**) Dimension-reduction and clustering of HCC tumors. Dimension-reduction was performed using Uniform Manifold Approximation and Projection (UMAP) algorithm, and clustering was performed using Louvain algorithm. (**b**) Comparison of prognosis among patients in different subtypes. P-value and HR were based on C1 versus a pool of C0 and C2. (**c**) Somatic mutation landscape. (**d**) Total number of somatic mutations among tumors in different subtypes. P-value was calculated using Kruskal-Wallis test. (**e**) Comparison of the most frequently mutated genes among subtypes. P-values were calculated using Chi-squared tests. *: *p* < 0.05; **: *p* < 0.01; ***: *p* < 0.001

Besides the malignant level evaluation, molecular subtyping is also of high significance in clinical practice, as it can aid patient-specific treatments for precision medicine. Considering the mechanisms of HCC tumorigenesis are drastically different between Asian and Western countries [30], we picked up the HCC transcriptome data from Asian patients (*N* = 154) and conducted an unsupervised dimension-reduction and clustering analysis. As a result, the HCC tumor samples were categorized into three subgroups (which were defined as C0, C1, and C2 subtypes; Fig. 4a). No significant gender biases among the three subtypes were observed, while the clinical stage distributions were different among subtypes (*P* = 0.016, Kruskal-Wallis test); in the post-hoc analysis, we found that C1 subtype showed higher proportion of late-stage tumors compared to C0 subtype (*P* = 0.017, Dunn test; Suppl. Fig. S15b). In addition, samples in subtype C1 showed significantly higher SAHR values than the other two subtypes (Suppl. Fig. S15c); indeed, patients in C1 subtype showed significantly worse survival than the other two subtypes (Fig. 4b). The results suggested that our classification of HCC subtypes possessed clinical value worthwhile for further investigations.

We analyzed the somatic mutation profiles across the three HCC subtypes, which was independent of the transcriptome data. The total somatic mutations were no significant differences among subtypes (*P* = 0.051, Kruskal-Wallis test), while detailed investigation showed that in the top 20 most frequently mutated genes in HCC, 8 of them (*TP53, CTNNB1*, *PCLO*, *OBSCN, AXIN1*, *CSMD3*, *APOB*, and *CACNA1E*) showed significant variations among subtypes (Fig. 4e). For instances, more than 80% tumors in the C2 subtype suffered from *CTNNB1* mutation, while the mutation frequencies of *CTNNB1* were lower than 20% for the other two subtypes; in contrast, the mutation frequencies in *TP53* and *AXIN1* genes were significantly reduced in C2 subtype (Fig. 4e). These results suggested that differences in molecular level existed among the 3 HCC subtypes.

### Molecular signatures across HCC subtypes

**Fig. 5:**
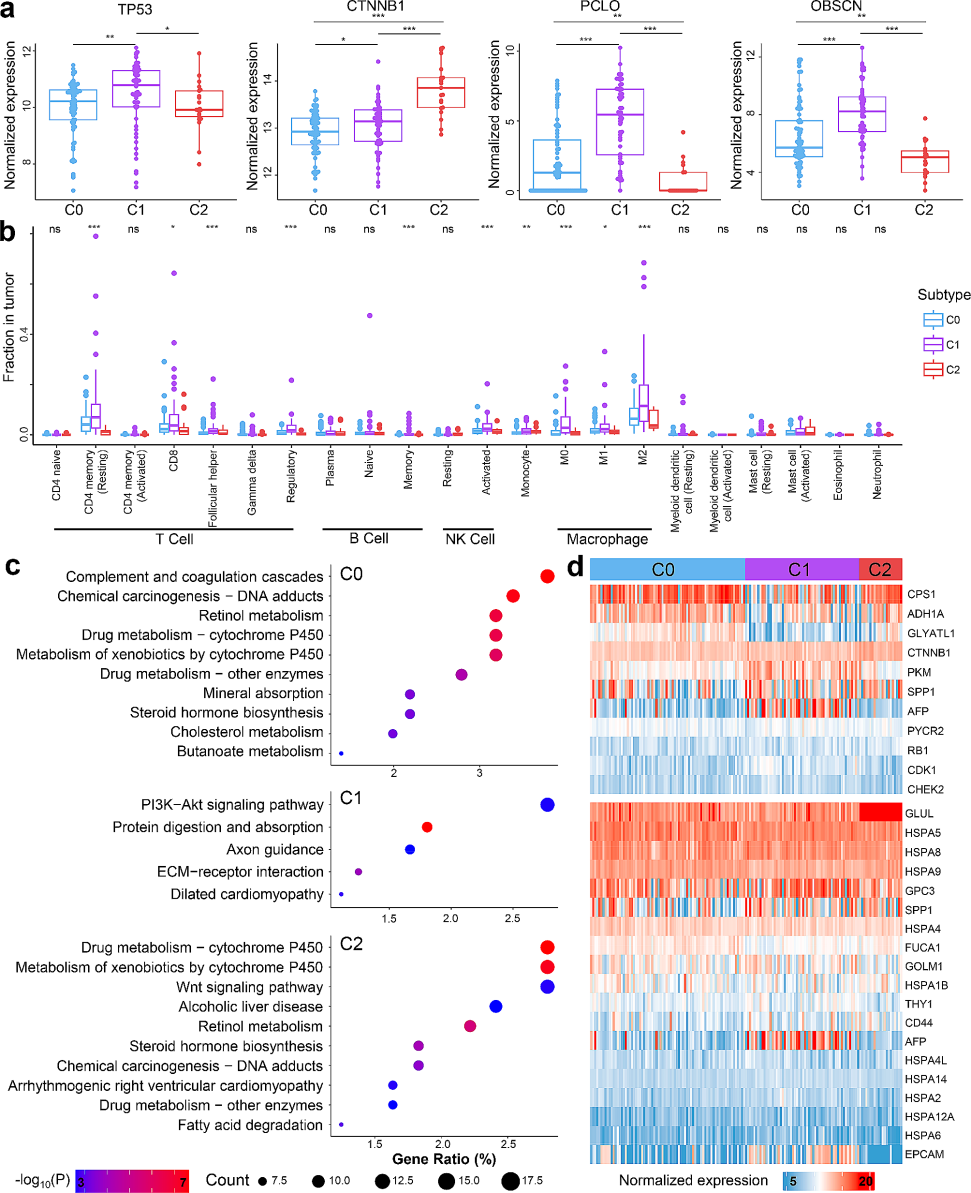
Functional annotation of HCC subtypes. (**a**) Illustration of differentially expressed genes among subtypes. P-values were calculated using Mann-Whitney U tests. (**b**) Illustration of immune infiltrations among subtypes. P-values were calculated using Kruskal-Wallis tests. (**c**) Enriched pathways for genes up-regulated in each subtype compared to the other subtypes. (**d**) Expression patterns of important genes reported in previous studies among subtypes. *: *p* < 0.05, **: *p* < 0.01, ***: *p* < 0.001

To explore the molecular signatures of the three HCC subtypes, differentially expressed genes for each subtype were mined by comparing them with the other two subtypes. As a result, thousands of DEGs were found for each subtype (Suppl. Fig. S16). Interestingly, we found that the expression levels of *CTNNB1* and *TP53* across the three subtypes were similar to their mutation frequencies (Fig. 5a), i.e., the C2 subtype showed higher expression level and higher mutation frequency of *CTNNB1* while lower expression level and lower mutation frequency of *TP53*; by contrast, expression patterns of *PCLO*, *OBSCN* and *CSMD3* were opposite to their mutation frequencies among the three subtypes (Fig. 5a and Suppl. Fig. S16b). Furthermore, proportions of various types of infiltrated immune cells differed significantly among the HCC subtypes (Fig. 5b and Suppl. Fig. S16c); notably, M2 macrophages were significantly higher in C1 subtype, whose infiltration was reported to correlate with metastasis and a poor prognosis in HCC patients [31] and suggested a more repressive immune environment in this subtype. In addition, CD4 memory and regulatory T cells were also significantly higher in C1 subtypes. Functional enrichments of the subtype specific DEGs were then investigated, which revealed various altered signaling pathways in each subtype (Fig. 5c and Suppl. Fig. S16d). For example, multiple metabolism related pathways were significantly altered among the three subtypes: up-regulated genes in C0 and C2 subtypes were enriched in retinol and drug metabolism pathways, while up-regulated genes in C1 subtype were enriched in PI3K-Akt and protein digestion and absorption pathway. A more detailed analysis showed that genes in plenty of metabolism pathways were down-regulated in C1 subtype, such as amino acid metabolism, fatty acid metabolism, and primary bile acid metabolism (Suppl. Fig. S16d). Moreover, a variety of biological processes were also altered: symporter activity, monooxygenase activity, and transmembrane receptor protein tyrosine kinase activity were the most enriched biological processes in C0, C1, and C2 subtypes, respectively (Suppl. Fig. S17).

To further explore the molecular alterations behind the poor prognosis of patients in C1 subtype, we investigated the expression patterns of 9 genes known to be highly important to HCC as well as 2 prognosis-related genes identified in Gao et al. using proteomics data [16]. As shown in Fig. 5d, subtype C1 showed significant differential expressions compared to the other two subtypes, *AFP*, *PKM*, and *CDK1* were up-regulated, while *CPS1* and *GLYATL1* were down-regulated; in addition, *ADH1A* genes down-regulated in C1 subtype. Furthermore, a list of 19 plasma biomarkers clinically used in non-invasive HCC diagnosis was also investigated [16] and the results were shown in Fig. 5d: *GLUL* was significantly up-regulated in C2 subtype, while *EPCAM* (epithelial cell adhesion molecule) and *AFP* were significantly up-regulated in C1 subtype, which results were concordant with a recent study [32] and suggested that abundance of these genes in plasma of HCC patients might inform their subtypes.

## Discussion

In this work, we performed an in-depth analysis on tumor transcriptomes through integration with patients’ prognoses. We showed that many genes were dysregulated and associated with patients’ survival across cancers in a cancer type-dependent manner, which allowed us to build models for tumor malignant level evaluation and molecular subtyping. In cancer, oncogenes and tumor suppressors are widely considered as the most important genes. Intuitively, the dysregulated oncogenes and tumor suppressors should play roles during carcinogenesis and therefore link to worse prognosis of the patients. However, investigations on the known oncogenes and tumor suppressors showed that the majority of them were not associated with patients’ prognoses; moreover, some of them showed diversified and inconsistent prognosis-associations with their annotations, which were consistent with previous reports on dual-functions of these genes [33, 34]. For instance, *HOXD11* is annotated as an oncogene in NCG database, while it showed multiple behaviors in patients’ survival (Fig. 2a). Functional studies revealed that *HOXD11* could enhance invasion, decompose the extracellular matrix, and epithelial mesenchymal transition-like phenotype metastasis through various downstream genes and pathways (e.g., JAM-A gene, NF-κB and FN1/MMP2/MMP9 signaling pathways) [35, 36]; however, it could also regulate the TGF-β signaling pathway to inhibit cell proliferation and cell cycle, i.e., functions like a tumor suppressor [37]. As another example, *PTEN* is a well-known tumor suppressor that negatively regulates cell proliferation, migration, and angiogenesis by antagonizing the PI3K-Akt/mTOR pathway [38, 39]; however, *PTEN* could also enhance PNCK-mediated-ERK1/2 inhibition to promote cellular proliferation, as well as cause FOXO-dependent upregulation of p53 suppressor Bcl6 and allow tumor cells to escape p53- and p21-mediated cellular senescence in leukemia [40]. On the other hand, the genes in NCG database had been functionally validated to play important roles in cancer, and their differential expressions and associations with prognosis could be affected by small sample size and other cofounders in cancers analyzed in this study. Considering the complexity and heterogeneity of cancers, our results suggested that for the cancer-related genes, their functions might not be as simple as an annotated label in the database and could be different across cancer types. Hence, investigations on the biological mechanisms of such cancer-related genes should be performed in a cancer-type-dependent manner.

In addition, we also screened genes that were frequently dysregulated and associated with patients’ survival, resulting in 45 genes discovered (42 of them were not annotated as oncogenes or tumor suppressors in the current database). Of note, some of the genes had been studied to infer their functions in cancer. As an example, *DUXAP8*, a long noncoding RNA that showed “oncogene-like” behavior in 5 cancer types, could recruit histone demethylase LSD1 and histone methyltransferase EZH2 to repress tumor suppressors [41, 42]; in addition, it could also suppress ferroptosis and induces drug resistance [43], thus functions in an “oncogene” manner. As another example, *DNASE1L3* gene behaves as “suppressor-like” in 5 cancer types. *DNASE1L3* is primarily known as an endonuclease that plays important roles in apoptotic DNA fragmentation [44–46]. Mechanism studies showed that *DNASE1L3* recruits cytoplasmic β-catenin destruction complex components (GSK-3β and Axin), promotes β-catenin ubiquitination and inhibits its nuclear translocation, thereby reducing c-Myc, P21 and P27 levels and negatively regulating proliferation, invasion and metastasis [47], supporting its “suppressor-like” behavior. Hence, these newly catalogued dysregulated and prognosis-associated genes could be valuable in cancer biology, and therefore deserve further functional investigations.

In clinical practice, tumor malignant level evaluation holds high significance in precision oncology. For example, high malignant level tumors suggest that the patients may not benefit too much from standard-of-care treatments. Conventionally, the tumor stages or grades were widely used in this task, where the molecular level information of the tumors is not taken into consideration. In this study, we developed a universal and quantitative measurement, i.e., the SAHR model, for evaluating the malignant level of tumors across cancers. We showed that the SAHR model worked well for all 13 cancer types in TCGA (Fig. 3 and Suppl. Fig S3-S13) and it was further validated using 2 non-TCGA datasets with a different transcriptome profiling platform (Fig. 3f-g); more importantly, in many cancer types, patients in similar stages with different SAHR values showed significantly distinct prognoses, suggesting that the SAHR model could add value to the clinical stage information, and these two parameters could be used in combinations for better healthcare of the patients. Compared to traditional TNM staging system that mostly based on size and spread of the tumor for malignant level estimation, our SAHR model leveraged the molecular information of the tumors. Considering the high heterogeneity in tumors, molecular level gene expression pattern might contain information of different dimension than morphology. In fact, for cancer patients of similar stages, SAHR values could still differentiate patients with different prognoses (Fig. 3h-k), suggesting that it could add value to the current staging system. Hence, we think that combination of TNM stage and SAHR value could be a preferable approach in clinical practice. The Fluorescence in situ hybridization (FISH) technology is also widely used in clinical practice, and such experiments may be performed on the genes utilized in our SAHR models for tumor malignancy level estimation and prognosis prediction. Moreover, tumor microenvironment is also relevant to patients’ prognoses [48–51]. In fact, various immune-related genes were utilized in our SAHR model (Suppl. Table S5). However, inference of immune cell infiltrations from bulk RNA-seq data is challenging [52], therefore integrating RNA-seq with other data on tumor microenvironment evaluation, such as immunohistochemistry staining and single-cell experiments [51], would be more appropriate to take advantage of the tumor environment in tumor malignancy level evaluation. On the other hand, currently SAHR should be considered as a proof-of-concept to model the tumor malignancy level, while more complex models could be built towards higher performances; however, it is challenging to fine-tune complex models with the limited sample size in hand, therefore it would be worthwhile trying in future studies with larger datasets.

In addition, we proposed a molecular subtyping approach for HCC, which revealed three distinct subtypes among Asian patients with different prognostic outcomes as well as various molecular-level discrepancies. Although we only used transcriptome data, our subtyping result was highly consistent with previous studies utilizing multi-omics and proteomics [16, 30]. For instance, subtype C0 showed up-regulated genes enriched in multiple metabolism-related pathways, including drug metabolism, acid metabolism and glucose metabolism, which was similar to the metabolism subgroup (named “S-Mb”) in Gao et al. [16]; subtype C1 highly assembled the “S-pf” (proliferation subgroup) HCC subtype defined in Gao et al. as they shared plenty of differentially expressed genes; subtype C2 has significantly higher mutation level in *CTNNB1* gene, higher expression level of *GLUL* and lower expression level of *EPCAM*, along with WNT pathway activation, which features are highly consistent with a specific HCC subtype reported in previous studies [53, 54]. Moreover, through functional annotations on the HCC subtypes, we found that genes for various metabolism pathways were down-regulated in the poorest prognostic HCC subtype (i.e., C1) compared to the other two subtypes, including glucose metabolism, energy generation via adenosine triphosphate (ATP), as well as amino acid and fatty acid metabolism. These alterations might enhance the tumor’s ability to thrive, proliferate, and metastasize, leading to poor survival of the patients [55]. For instance, most genes in the drug metabolism of cytochrome P450 pathway were significantly down-regulated in C1 subtype, which mediates the metabolic activation of numerous procarcinogens and participates in the inactivation and activation of anticancer drugs [56] and associates with fast-growing HCC and worse prognosis [57]. Moreover, the primary bile acid biosynthesis pathway also varies among subtypes. Bile acids are critical regulators of the development of various liver diseases [58], and higher levels of glycine and taurine conjugated primary bile acids were associated with a 2- to 8-fold increased risk of HBV- and HCV-related HCC [59]. The immune infiltration landscape also differed among the HCC subtypes (Fig. 5b and Suppl. Fig. S15c). For example, the C1 subtype has a higher proportion of M2 macrophage, which is known to associate with metastasis and poor prognosis in HCC patients [31]. Hence, tumor subtyping could also enhance our understanding of the underlying mechanisms that contribute to the heterogeneity and progression of the tumors [32]. Notably, compared with previous studies, our approach only utilized the transcriptome data, which was easy to obtain with low experimental complexity and cost, suggesting that our approach might possess certain advantages in clinical promotion.

In summary, through integrative analysis of tumor transcriptome and patient’s prognosis, we proposed easy-to-perform models aiming to improve the living condition of cancer patients towards precision oncology.

## Methods

### Data resources and curation

Transcriptome and clinical data were obtained from TCGA and GEO under accession numbers GSE17538 [28] and GSE31210 [29]. To achieve statistical power in downstream analyses, major cancer types with more than 100 tumors and 10 adjacent normal tissues were kept (*N* = 13; Suppl. Table S1). For BLCA, tumors annotated as “Papillary adenocarcinoma” or “Squamous cell carcinoma” were filtered out due to limited sample size, and only “Papillary transitional cell carcinoma” or “Transitional cell carcinoma” were kept. For BRCA, the infiltrating duct and lobular carcinoma were two major subtypes of BRCA, but in the subtype of lobular carcinoma, only 4 adjacent normal samples were available, so only the “Infiltrating duct carcinoma” subtype was kept. For COAD, only tumors annotated as “Adenocarcinoma” were kept. For HCC, only included the tumors annotated as “Hepatocellular carcinoma”. Tumors of LC and TC were both selected from head and neck squamous cell carcinoma (HNSC) and treated as 2 cancer types. We studied laryngeal cancer and tongue cancer separately due to the tumors come from different tissues. For KIRC and KIRP, only tumors annotated as “Clear cell adenocarcinoma” or “Papillary adenocarcinoma” were kept, respectively. For LUAD, tumors annotated as “Adenocarcinoma” or “Adenocarcinoma with mixed subtypes” were kept. For LUSC, tumors annotated as “Squamous cell carcinoma” were kept. For STAD, tumors annotated as “Adenocarcinoma intestinal type” or “Adenocarcinoma” were kept. For THCA, tumors annotated as “Papillary adenocarcinoma” or “Papillary adenocarcinoma” were kept. For UCEC, tumors annotated as “Endometrioid adenocarcinoma” were kept.

### Differential expression analysis

For each cancer type, we used the raw read counts (provided by TCGA) from all the tumors and adjacent normal to mine the differentially expressed genes using DESeq2 software [60]; genes with Benjamini-Hochberg adjusted p-values lower than 0.01 and at least 2-fold expression changes between tumors and adjacent normal tissues were considered as differentially expressed [61]. Ribosomal RNA genes were removed from the analysis. Note that for each cancer type, DESeq2 would automatically normalize the expression of all the genes based on a negative binomial model, and the normalized values were used in the downstream expression-related plots.

### Survival analysis

For each cancer type, we randomly split the tumor samples into training and testing datasets (Suppl. Table S1). For each gene of interest in a specific cancer type, we first calculated the median expression value in the tumor samples of the training dataset then split the samples into 2 groups using the median expression value: the group whose expression was higher than the median value was named “higher expression” and the other was named “lower expression”. Note that some genes were not expressed (i.e., with an expression value of 0) in a substantial proportion of tumors and were handled slightly differently: if a gene was not expressed in more than 30% of all tumors, then tumor samples that did not express this gene would be grouped as “lower-expression” and the rest samples would be grouped as “higher-expression”. We then performed Kaplan-Meier analysis to compare the patients in the two groups and genes with P-values < 0.05 (obtained from univariable Cox regression) were considered as prognosis-related.

### Classification of prognosis associated genes

We explored the prognosis-associated genes for each cancer type separately. For each DEG mined in the differential expression analysis, if its overall expression was higher in the tumors than the adjacent normal samples, it would be considered as up-regulated; otherwise, the gene would be considered as down-regulated. For the up-regulated expressed DEG, if it was prognosis-associated in the survival analysis, we checked the HR of the “lower expression” versus “higher expression” groups of patients: the gene would be defined as “oncogene-like” if the HR was smaller than 1 (i.e., higher-expression linked to poorer patients’ survival; Fig. 1c), otherwise, it would be defined as “up-regulated-saver” (Fig. 1d). For the down-regulated DEG, if it was prognosis-associated, it would be defined as “down-regulated-saver” if the HR of the “lower expression” versus “higher expression” groups of patients was smaller than 1 (i.e., lower expression linked to better survival of the patients; Fig. 1e), otherwise it would be defined as “suppressor-like” (Fig. 1f).

### SAHR calculation

The SAHR is defined as a weighted accumulation of HRs of the four types of prognosis-associated genes defined in Fig. 3a. For each cancer type, 20, 20, 10 and 10 prognosis-genes with the most significant P-values were picked up from the “oncogene-like”, “suppressor-like”, “up-regulated-saver”, and “down-regulated-saver” categories to form a 60-gene list (genes with abnormally high HRs, i.e., > 20, were omitted). Then expression cutoffs (i.e., median expressions of the tumors in the training dataset) and HRs were extracted from the expression data and survival analysis (note that due to different behaviors in survival analysis for the 4 types of genes, we used the patient group with worse survival versus the other with better survival, e.g., “higher expression” group versus “lower expression” group for “oncogene-like” gene, to calculate HRs). For each sample in the testing dataset, the coefficient of each gene in the 60-gene list was extracted and a coefficient was assigned based on its expression and prognosis-association category: for “oncogene-like” genes, if its expression was higher than the cutoff, then “+1” would be assigned, otherwise “-1” would be assigned; for “up-regulated-saver” genes, if its expression was higher than the cutoff, then “-1” would be assigned, otherwise “+1” would be assigned; for “suppressor-like” genes, if its expression was higher than the cutoff, then “-1” would be assigned, otherwise “+1” would be assigned; for “down-regulated-saver” genes, if its expression was higher than the cutoff, “+1” would be assigned, otherwise “-1” would be assigned. Then SAHR was calculated using the following formula:

$$SAHR=\sum {\delta }_{i}\times {HR}_{i}$$

where *δ* denotes the coefficient defined above.

For each cancer type, we calculated the SAHR values for samples in their corresponding testing dataset; we then divided the samples into SAHR positive (i.e., SAHR value > 0) and SAHR negative (i.e., SAHR value < 0) groups to compare the prognoses of the patients between these two groups. To investigate the synergy between SAHR and clinical stage, due to the limited sample size, we split the patients into early (i.e., stage I or II) and late (i.e., stage III or IV) stages based on their clinical information; for each stage, we divided the samples into SAHR positive and SAHR negative based on the SAHR scores then compared the prognoses between these two groups.

### Subtyping of HCC tumors

HCC tumors from Asian patients (*N* = 154) were selected for subtyping analysis using the Seurat package [62]. We extracted the raw counts for each tumor sample and performed Log-Normalization [63] on the data. Then, the mean variance analysis was carried out and 2000 genes with high variations among the samples were screened out. Based on the expression levels of these highly variable genes, principal component analysis (PCA) was performed and the first 30 principal components were subjected to dimension reduction using the UMAP algorithm [64]; then Louvain algorithm was used for unsupervised clustering and each cluster was considered as a subtype. Kaplan-Meier analysis was performed on patients grouped by subtypes to investigate the prognosis differences among the subtypes. For all tumors, somatic mutations called by Mutect algorithm [65] were downloaded from TCGA, and MAFtools package [66] was used to perform mutation burden analysis and data visualization.

### Annotation of HCC subtypes

For each subtype, we mined the differentially expressed genes by comparing the transcriptomes of the tumors in this subtype versus all the rest samples using DESeq2 software [60]; genes with Benjamini-Hochberg adjusted p-values lower than 0.01 and showed at least 2-fold changes in expression were considered as differentially expressed. For the mined DEGs in each subtype, functional enrichments were performed using DAVID webserver [67, 68] against KEGG pathways and Gene Ontology (including Biological Process, Cellular Component, and Molecular Function), and enriched items with Benjamin-Hochberg adjusted p-values < 0.05 were considered as statistically significant. Gene set variation analysis (GSVA) was also performed to identify the altered pathways in each HCC subtype. Functional annotations were downloaded from the Molecular Signatures Database (MSigDB, the “c2.cp.kegg.v2023.1.Hs.symbols.gmt” file was used). The significance of the pathway enrichment scores in GVSA was estimated by linear model and moderated with F-statistic; pathways with Benjamin-Hochberg adjusted p-value < 0.05 were considered as differentially enriched. Immune infiltration analyses were performed with CIBERSORT (the ABS mode was used; Fig. 5b) [69] and ssGSEA software (Suppl. Fig. S16c) [70].

### Electronic supplementary material

Below is the link to the electronic supplementary material.

Supplementary Material 1

Supplementary Material 2

Supplementary Material 3

## Data Availability

The molecular and clinical data is obtained from TCGA. All scripts used in this work are publicly available at Github (https://github.com/hellosunking/tumor-transcriptome).
