# Supplementary material for: Generalizable transcriptome-based tumor malignant level evaluation and molecular subtyping towards precision oncology
Source: J Transl Med. 2024 May 28;22:512. doi: 10.1186/s12967-024-05326-0 (PMC11134716; doi:10.1186/s12967-024-05326-0)
Supplement: Supplementary file 2 — Supplementary Material 2 [file 12967_2024_5326_MOESM2_ESM.pdf]

Generalizable transcriptome-based tumor malignant level evaluation and molecular subtyping towards precision oncology

Dingxue Hu *et al.*

This supplementary file includes Suppl. Fig. S1-S17.

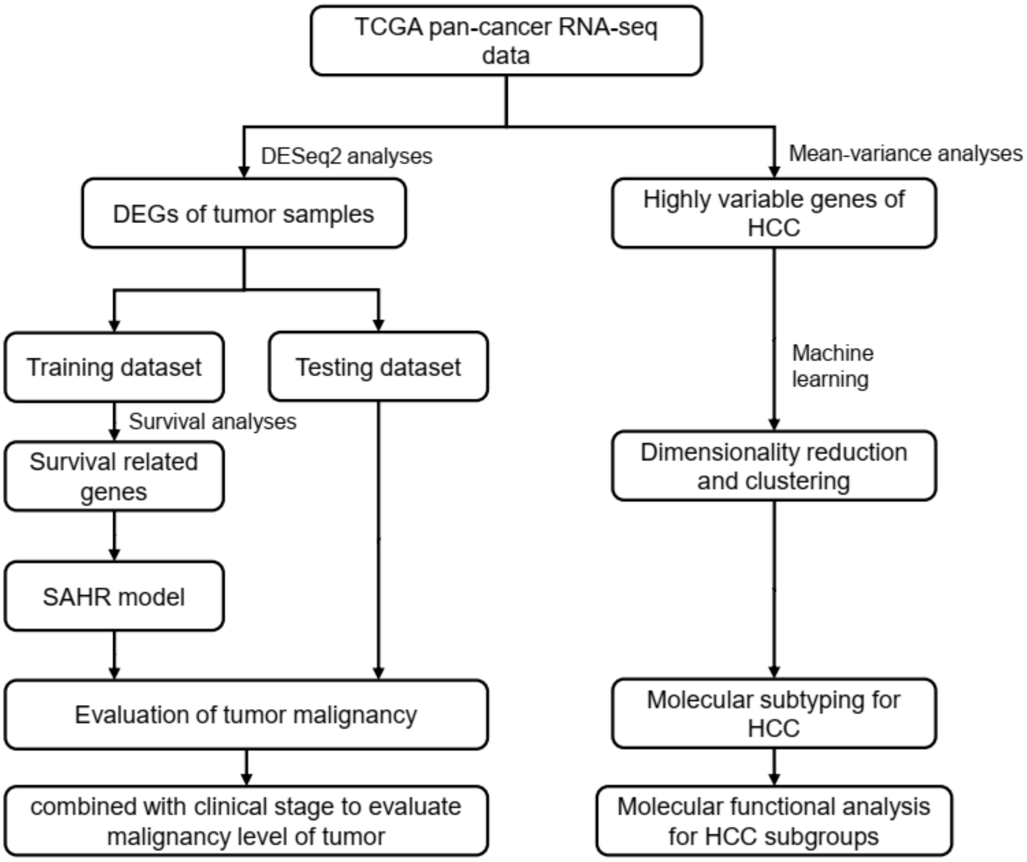

Fig. S1. Workflow of the analysis in this study.

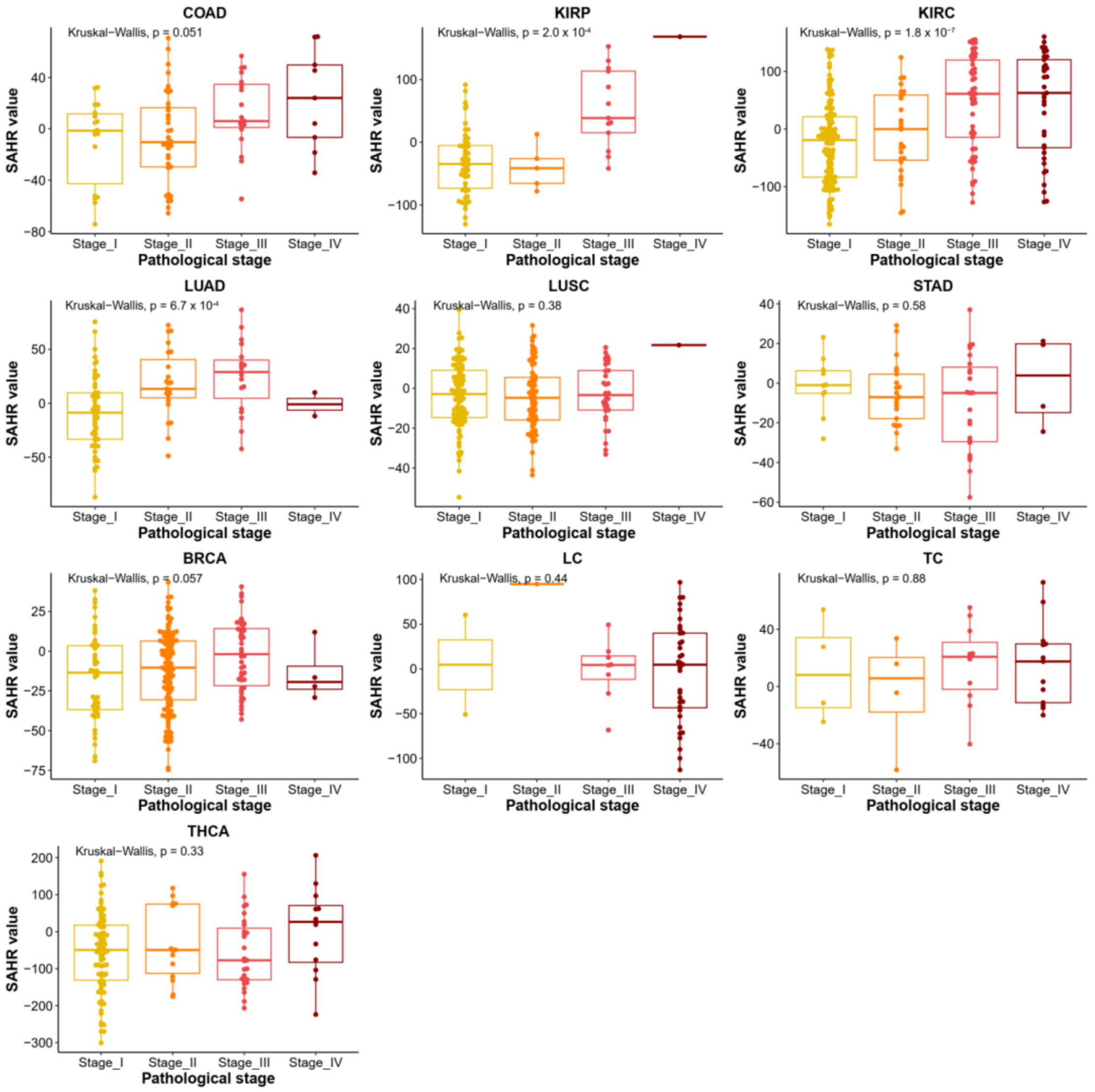

Fig. S2. Associations between SAHR value and clinical stage among various cancer types.

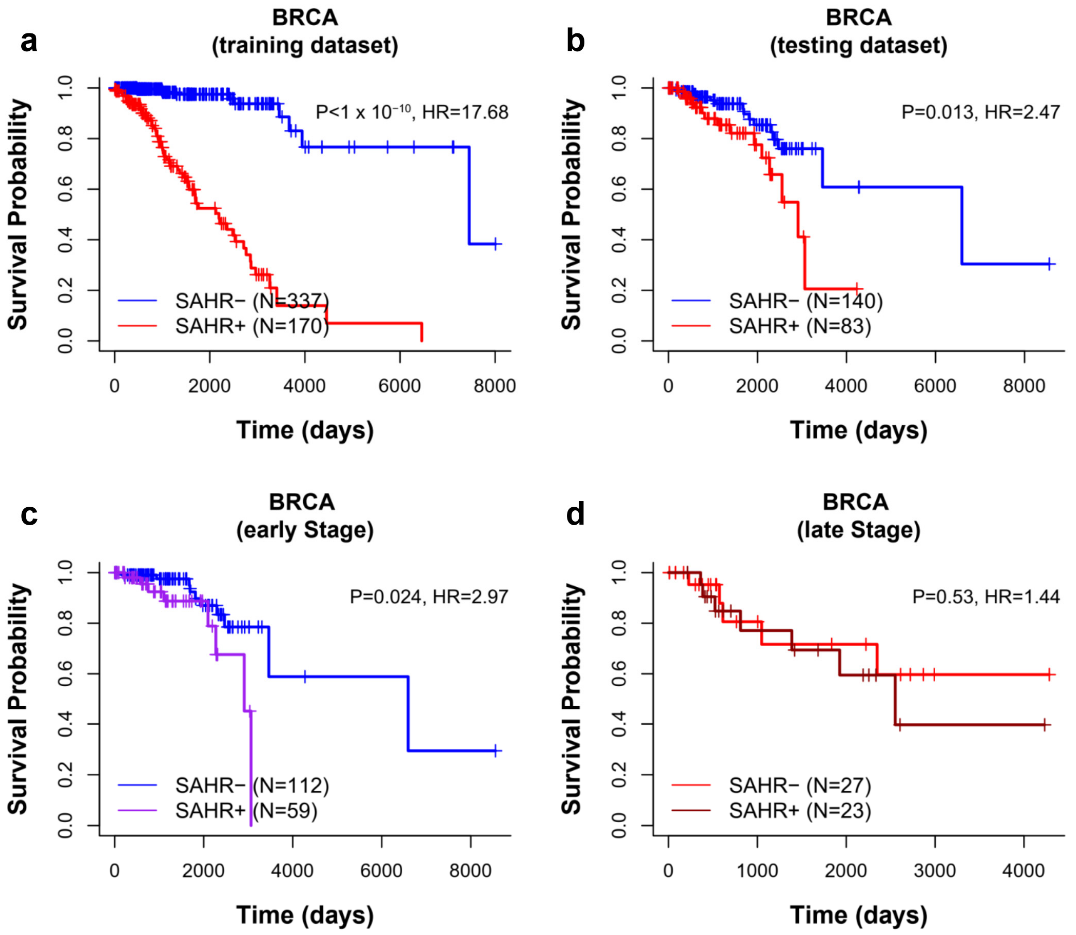

Fig. S3. Comparison of breast cancer (BRCA) patients with different SAHR values.

(a) training dataset, (b) testing dataset, (c) early- and (d) late-stage patients in testing dataset.

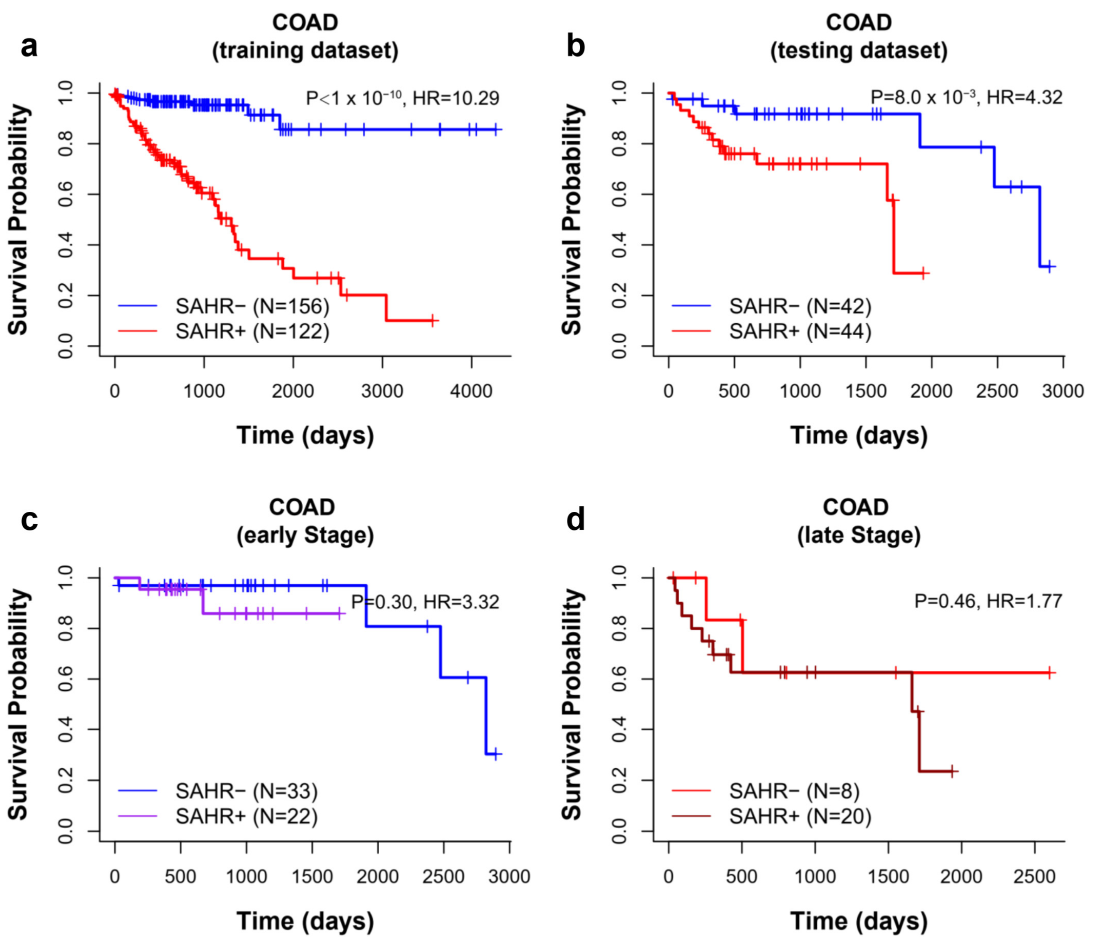

Fig. S4. Comparison of colon adenocarcinoma (COAD) patients with different SAHR values. (a) training dataset, (b) testing dataset, (c) early- and (d) late-stage patients in testing dataset.

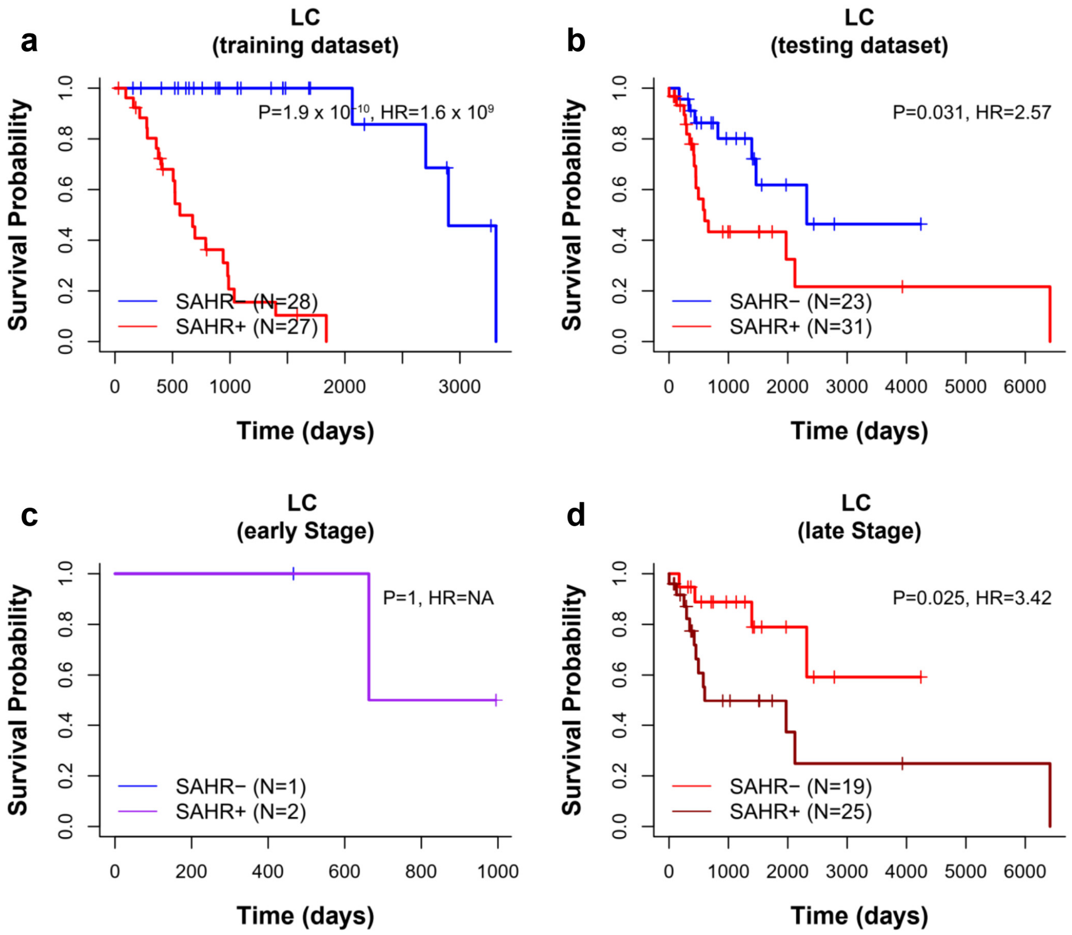

Fig. S5. Comparison of Laryngeal cancer (LC) patients with different SAHR values. (a) training dataset, (b) testing dataset, (c) early- and (d) late-stage patients in testing dataset.

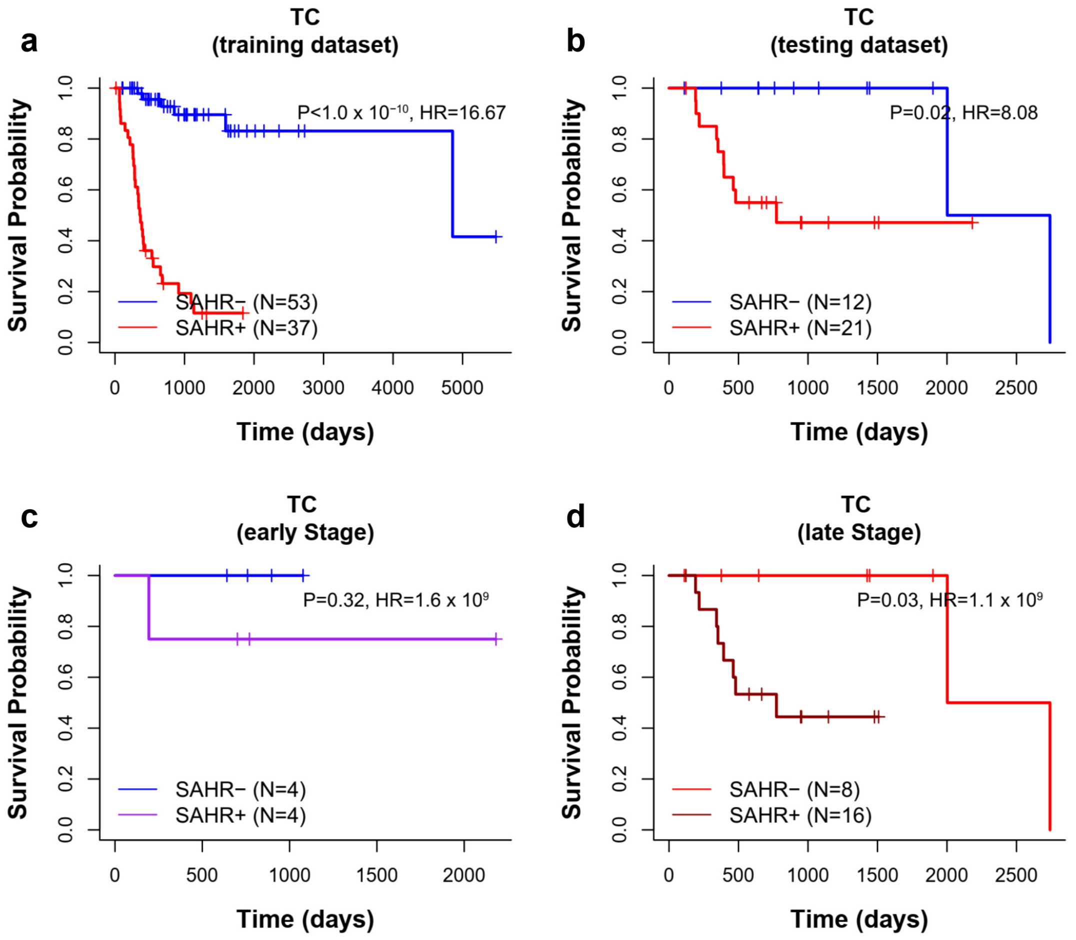

Fig. S6. Comparison of Tongue cancer (TC) patients with different SAHR values. (a) training dataset, (b) testing dataset, (c) early- and (d) late-stage patients in testing dataset.

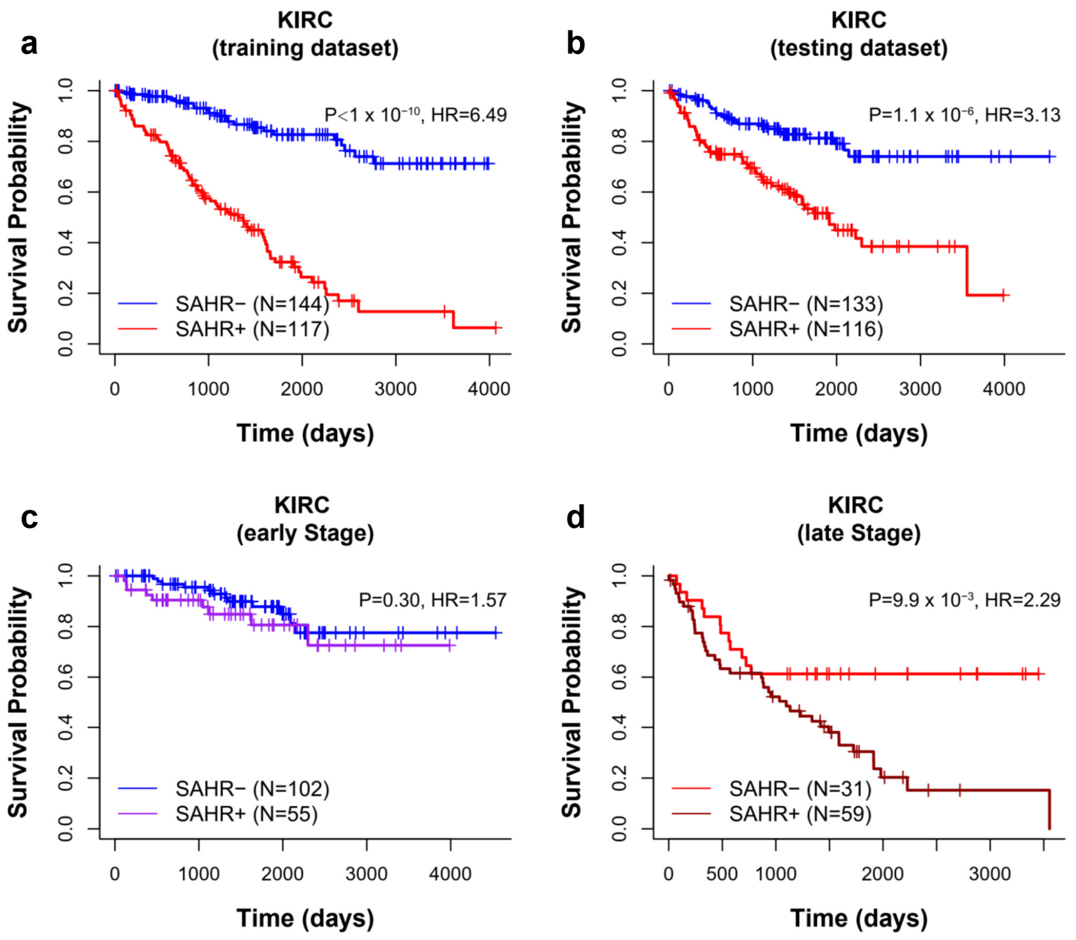

Fig. S7. Comparison of Kidney renal clear cell carcinoma (KIRC) patients with different SAHR values. (a) training dataset, (b) testing dataset, (c) early- and (d) late-stage patients in testing dataset.

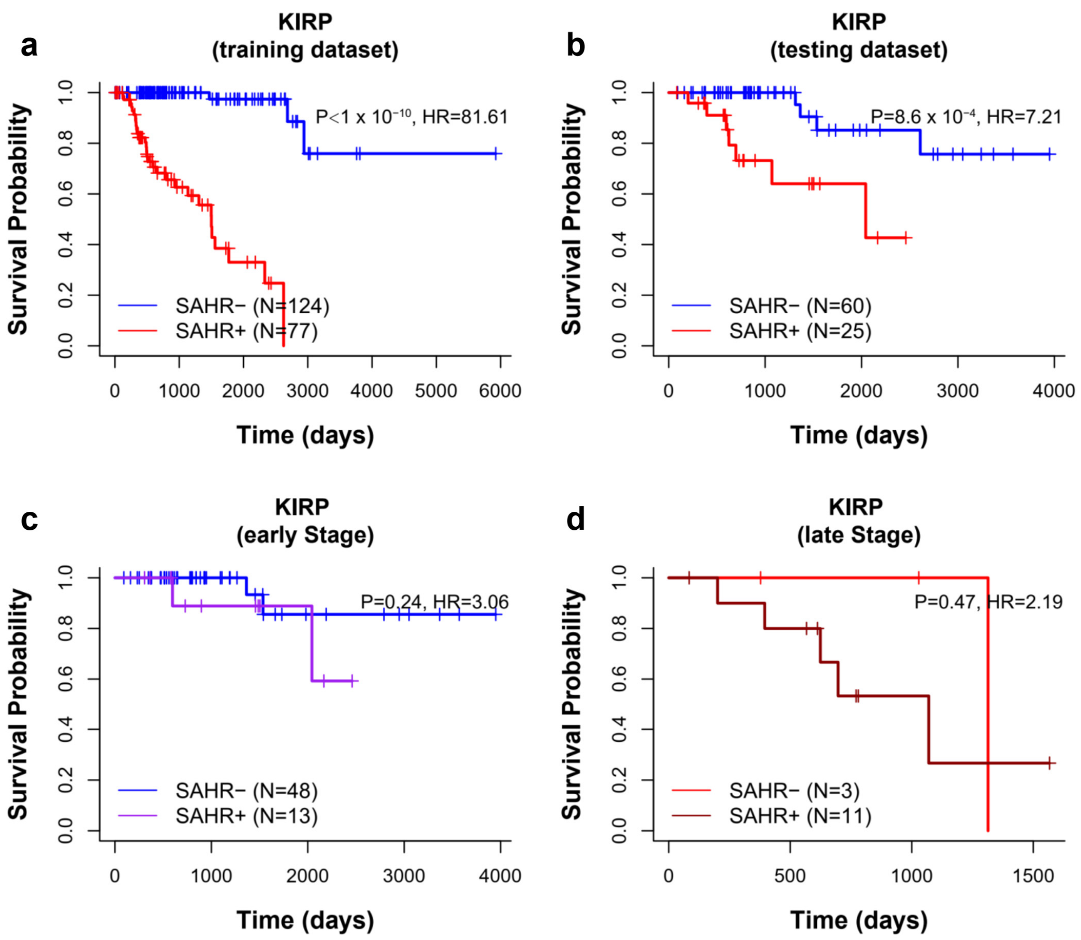

Fig. S8. Comparison of Kidney renal papillary cell carcinoma (KIRP) patients with different SAHR values. (a) training dataset, (b) testing dataset, (c) early- and (d) late-stage patients in testing dataset.

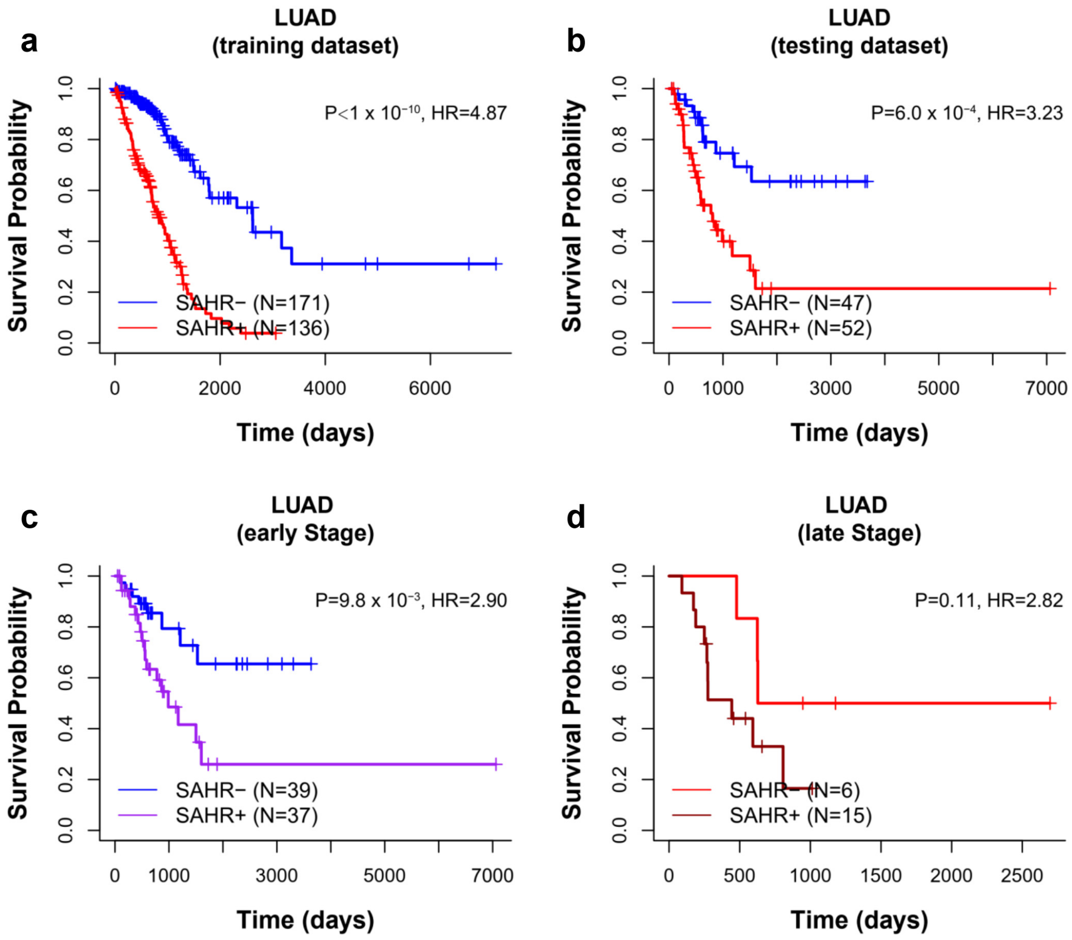

Fig. S9. Comparison of Lung adenocarcinoma (LUAD) patients with different SAHR values. (a) training dataset, (b) testing dataset, (c) early- and (d) late-stage patients in testing dataset.

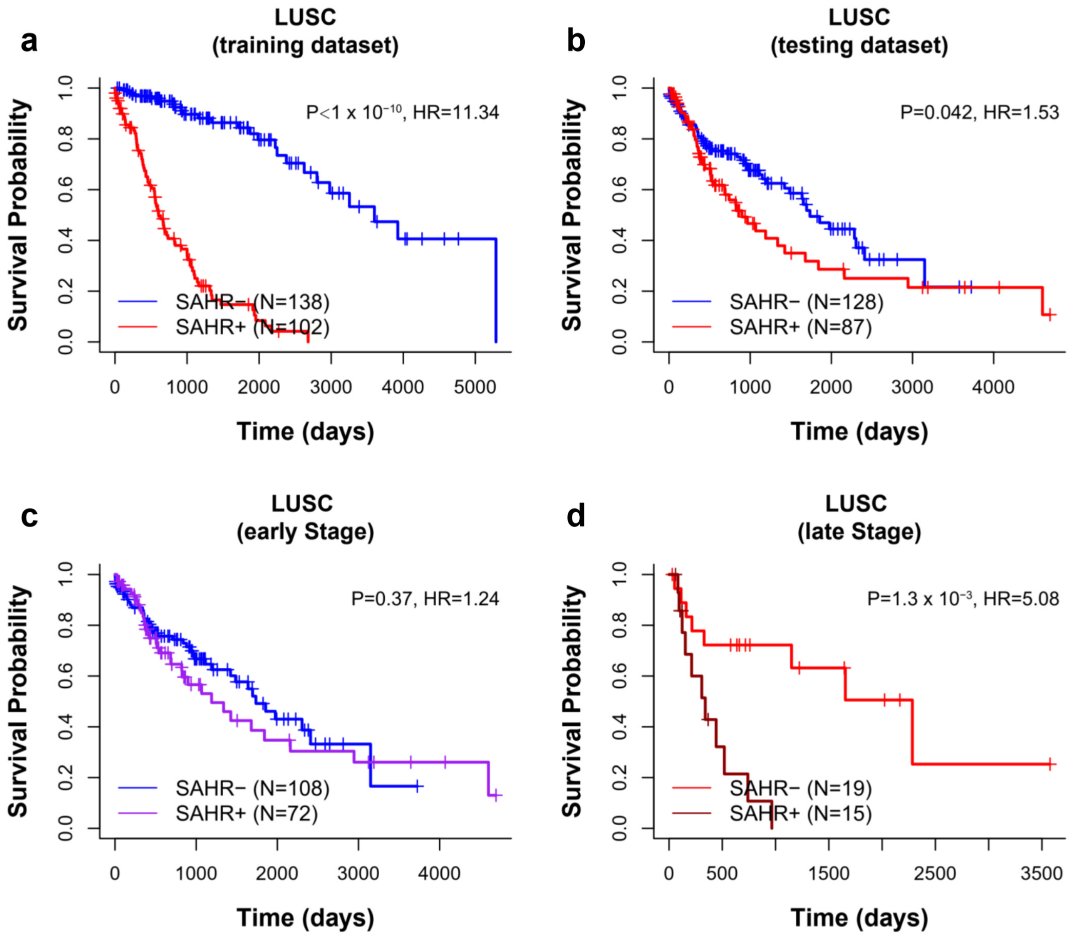

Fig. S10. Comparison of Lung squamous cell carcinoma (LUSC) patients with different SAHR values. (a) training dataset, (b) testing dataset, (c) early- and (d) late-stage patients in testing dataset.

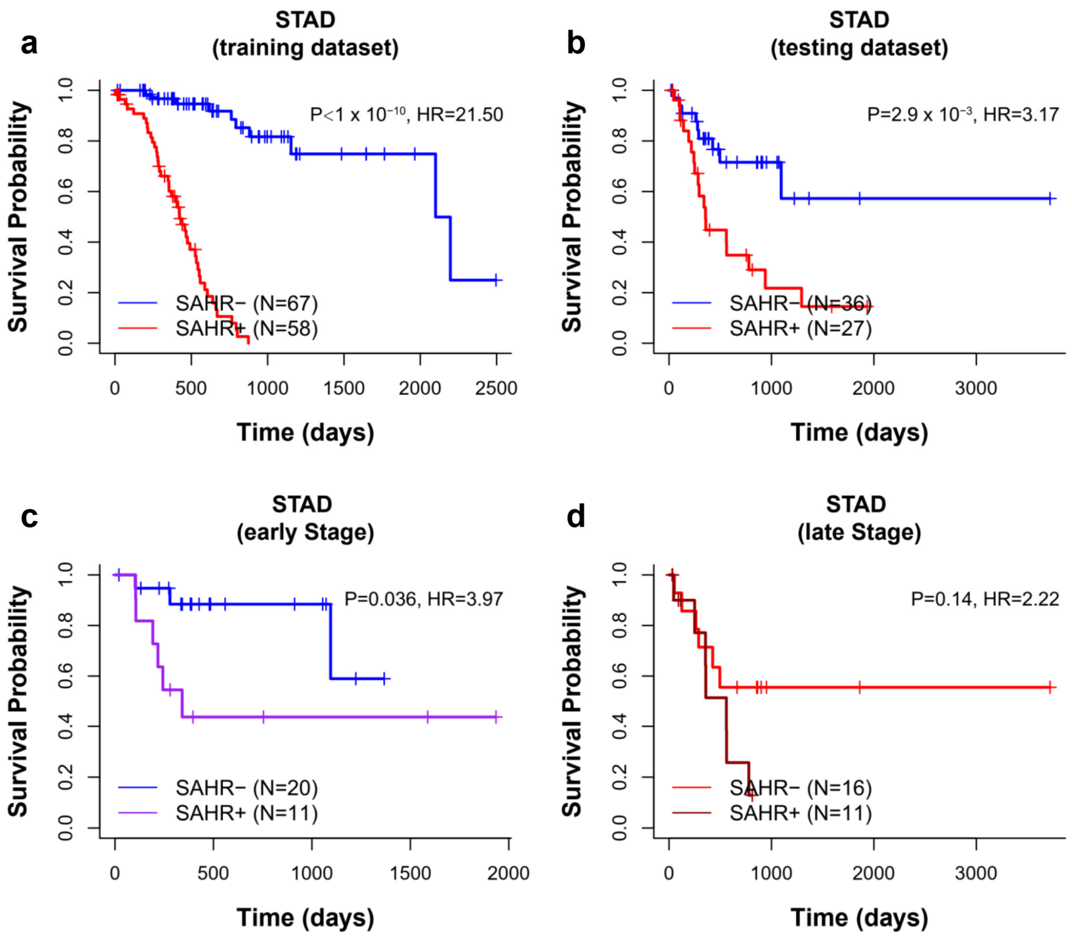

Fig. S11. Comparison of Stomach adenocarcinoma (STAD) patients with different SAHR values. (a) training dataset, (b) testing dataset, (c) early- and (d) late-stage patients in testing dataset.

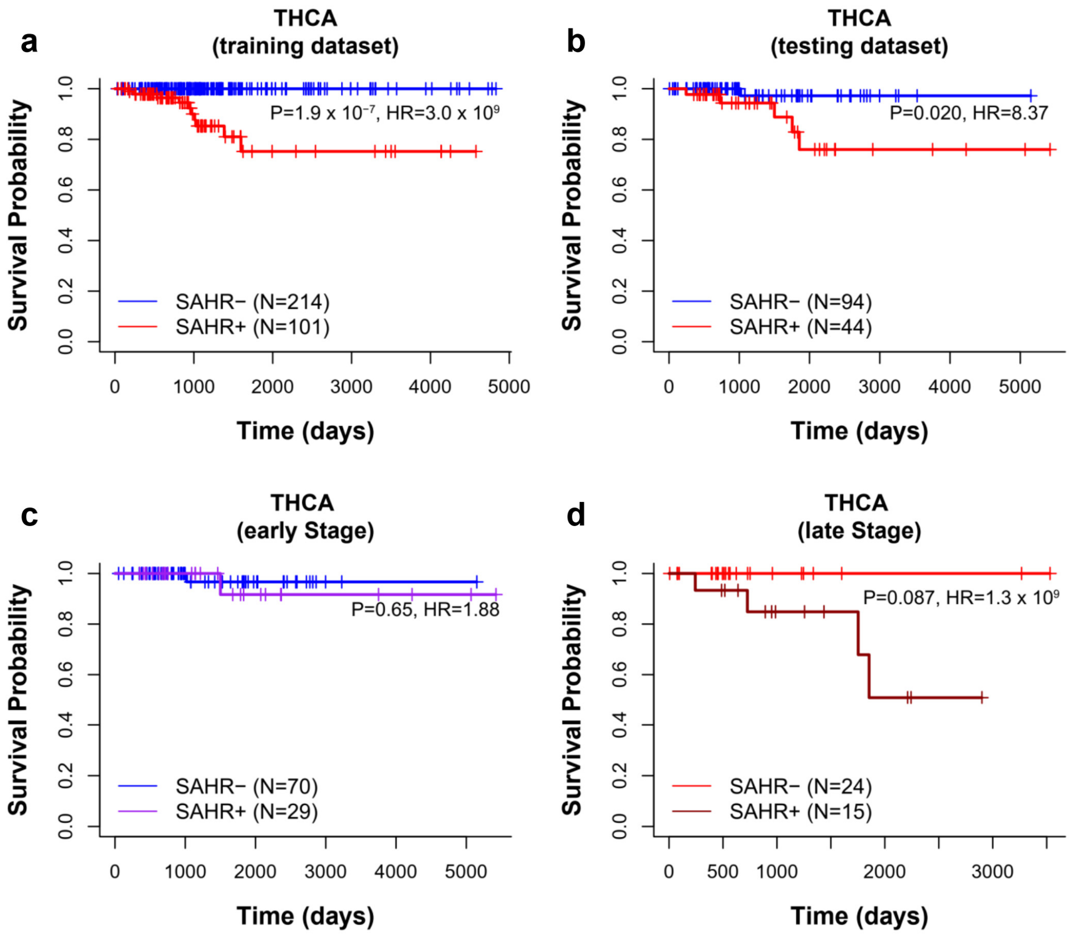

Fig. S12. Comparison of Thyroid carcinoma (THCA) patients with different SAHR values. (a) training dataset, (b) testing dataset, (c) early- and (d) late-stage patients in testing dataset.

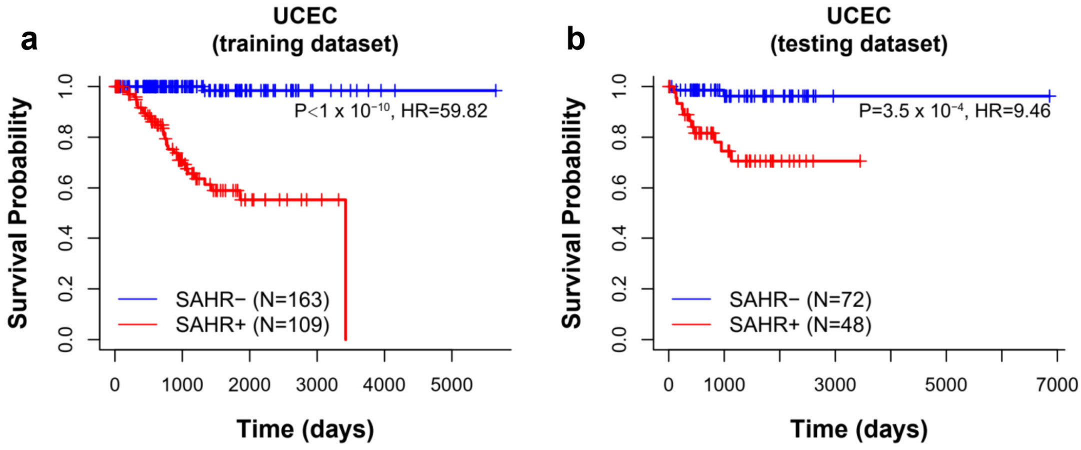

Fig. S13. Comparison of Endometrial endometrioid adenocarcinoma (UCEC) patients with different SAHR values. (a) training dataset, (b) testing dataset.

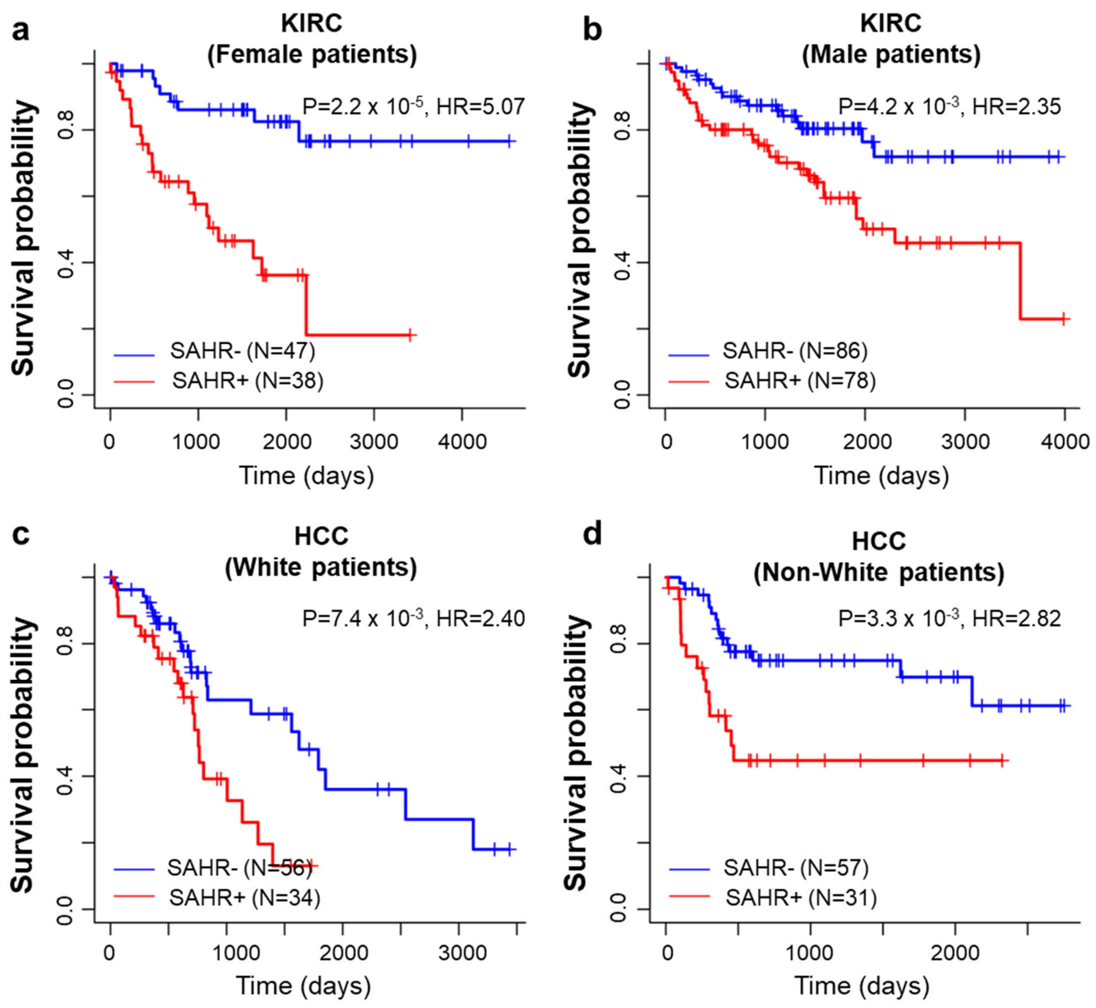

Fig. S14. Comparison of patients' survival for SAHR values in patients with different gender or ethics. (a) female and (b) male in KIRC, (c) White and (d) non-White in HCC.

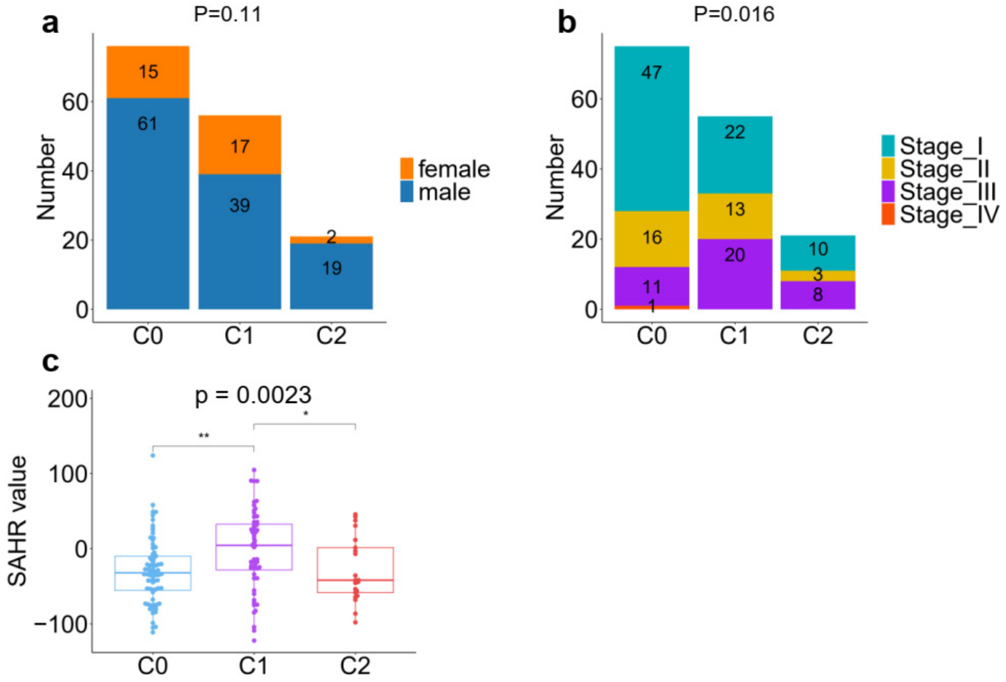

Fig. S15. Gender and clinical stage distribution of HCC patients in different HCC subtypes. (a) Gender, (b) Stage. P-values were calculated using Kruskal-Wallis tests.

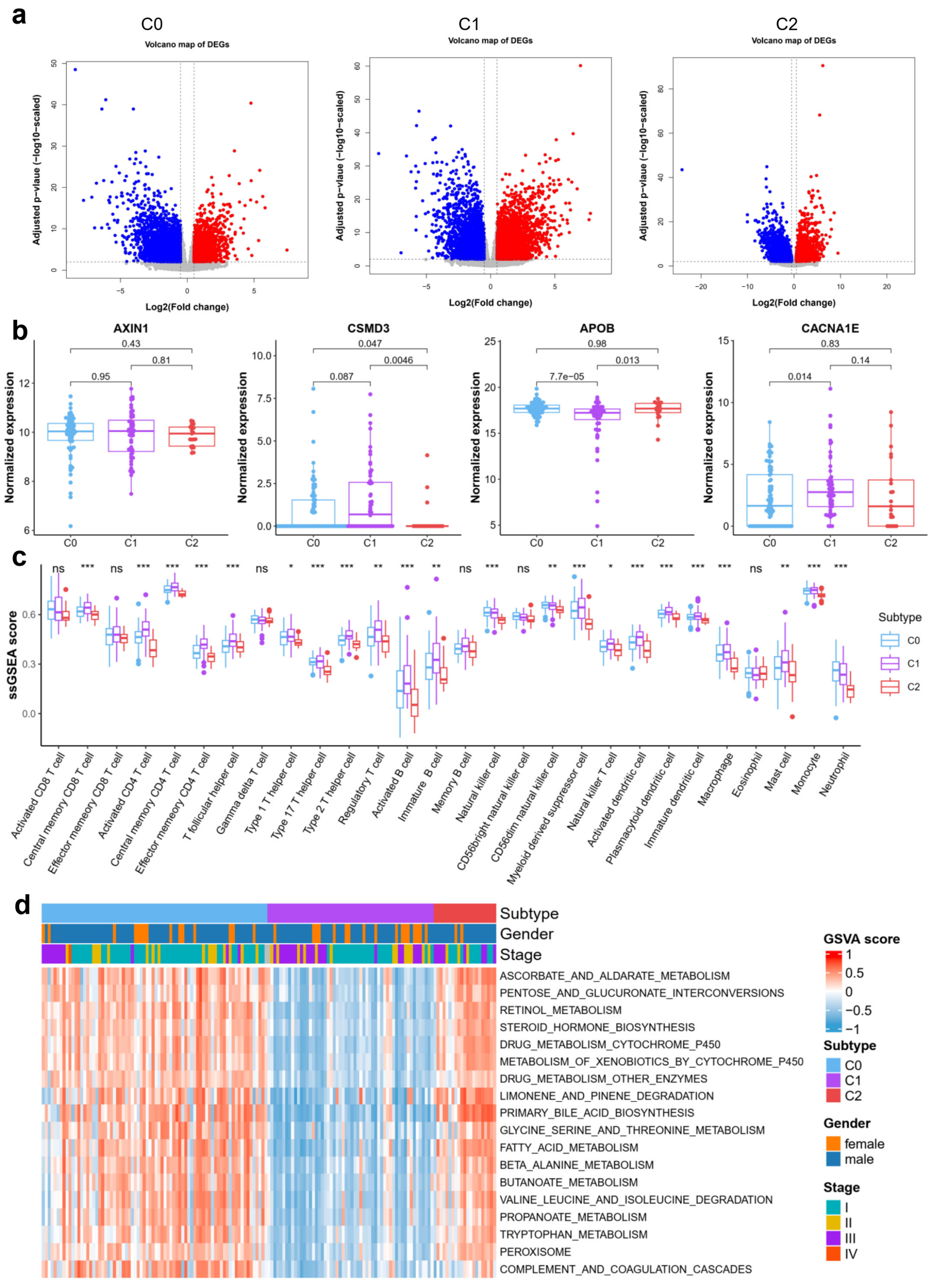

Fig. S16. Annotations for HCC subtypes. (a) Overall view of differentially expressed genes among HCC subtypes. (b) Expression levels for somatic mutation related genes (*AXIN1*, *CSMD3*, *APOB* and *CACNA1E*). (c) Immune infiltration landscape among HCC subtypes using ssGSEA algorithm. P-values were calculated using Kruskal-Wallis

tests, *: $p<0.05$, **: $p<0.01$, ***: $p<0.001$. (d) KEGG pathway enrichment analyses based on gene expression profile data using gene set variation analysis.

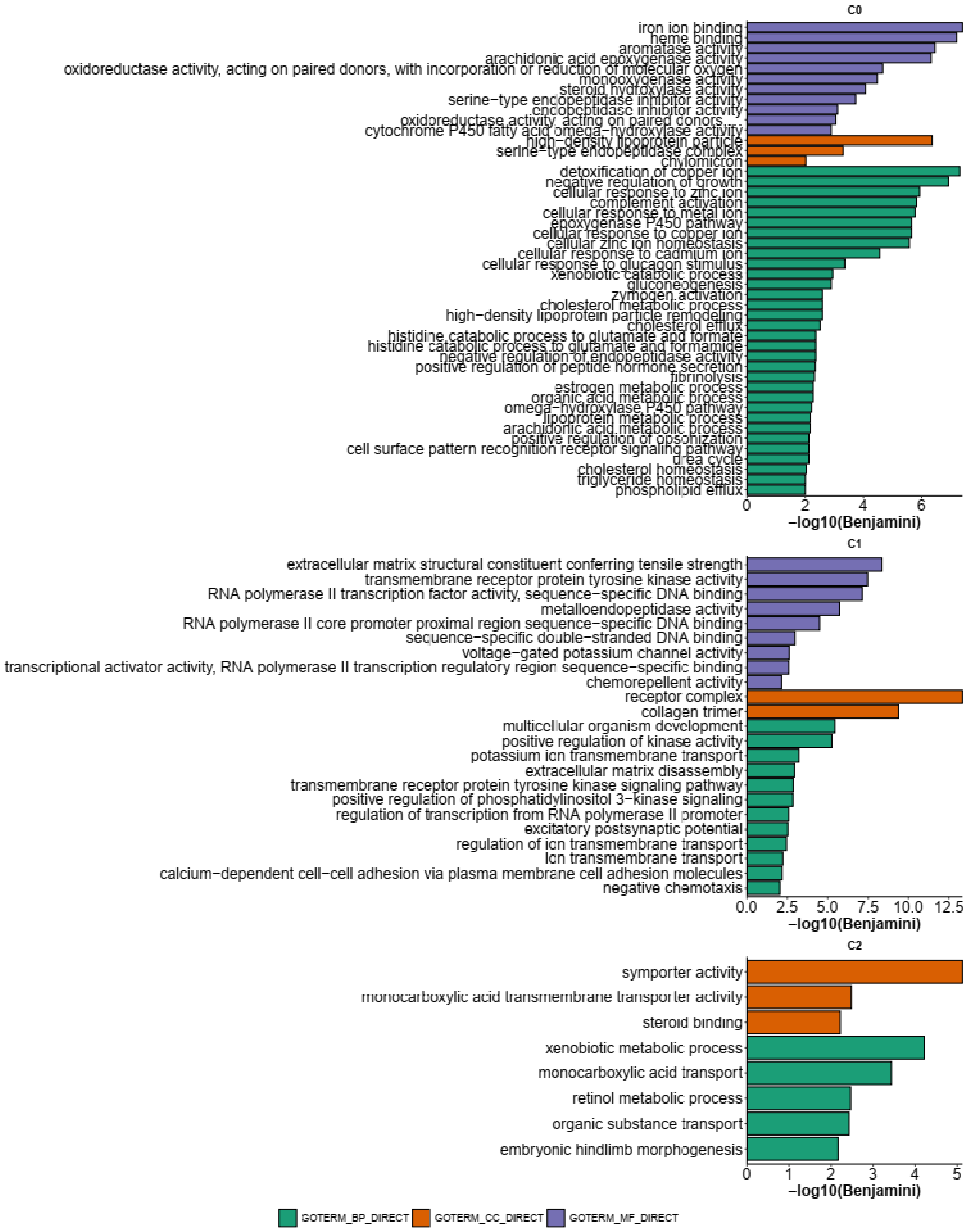

Fig. S17. Gene Ontology enrichment for the subtype-specific up regulated genes in HCC.
