# Supplementary material for: Generalizable transcriptome-based tumor malignant level evaluation and molecular subtyping towards precision oncology
Source: J Transl Med. 2024 May 28;22:512. doi: 10.1186/s12967-024-05326-0 (PMC11134716; doi:10.1186/s12967-024-05326-0)
Supplement: Supplementary file 3 — Supplementary Material 3 [file 12967_2024_5326_MOESM3_ESM.docx]

Dr. Francesco M. Marincola

Editor-in-Chief

*Journal of Translational Medicine*

May 8, 2024

Dear Dr. Marincola,

Thank you very much for your kind handling of our manuscript “*Generalizable transcriptome-based tumor malignant level evaluation and molecular subtyping towards precision oncology*” (JTRM-D-24-01967) and the opportunity of further revising it.

We are encouraged that most of the reviewers were satisfied with our revision. We have further revised the manuscript in accordance with the reviewers’ comments. The revisions have been detailed in the point-by-point responses and are also highlighted in the revised manuscript. We believe that the revised manuscript has been improved and hope that it would meet the criteria of acceptance for publication in *Journal of Translational Medicine.* We greatly appreciate your consideration.

Sincerely,

Kun Sun, Ph.D.

Assistant Professor

Shenzhen Bay Laboratory
